# Mentoring New and Early-Stage Investigators and Underrepresented Minority Faculty for Research Success in Health-Related Fields: An Integrative Literature Review (2010–2020)

**DOI:** 10.3390/ijerph18020432

**Published:** 2021-01-07

**Authors:** Lynda B. Ransdell, Taylor S. Lane, Anna L. Schwartz, Heidi A. Wayment, Julie A. Baldwin

**Affiliations:** 1 Center for Health Equity Research, Northern Arizona University, 1395 S Knoles Drive, Suite 140, Flagstaff, AZ 86011, USA; Taylor.Lane@nau.edu (T.S.L.); Anna.Schwartz@nau.edu (A.L.S.); Heidi.Wayment@nau.edu (H.A.W.); Julie.Baldwin@nau.edu (J.A.B.); 2 Department of Health Sciences, Northern Arizona University, 1100 S Beaver St, Flagstaff, AZ 86011, USA; 3 School of Nursing, Northern Arizona University, 202 E Pine Knoll Dr, Flagstaff, AZ 86011, USA; 4 Department of Psychological Sciences, Northern Arizona University, 1100 S Beaver St, Flagstaff, AZ 86011, USA

**Keywords:** early career, faculty development, underrepresented minority faculty, new faculty, diversity

## Abstract

Mentoring to develop research skills is an important strategy for facilitating faculty success. The purpose of this study was to conduct an integrative literature review to examine the barriers and facilitators to mentoring in health-related research, particularly for three categories: new investigators (NI), early-stage investigators (ESI) and underrepresented minority faculty (UMF). PsychINFO, CINAHL and PubMed were searched for papers published in English from 2010 to 2020, and 46 papers were reviewed. Most papers recommended having multiple mentors and many recommended assessing baseline research skills. Barriers and facilitators were both individual and institutional. *Individual barriers* mentioned most frequently were a lack of time and finding work–life balance. UMF mentioned barriers related to bias, discrimination and isolation. *Institutional barriers* included lack of mentors, lack of access to resources, and heavy teaching and service loads. UMF experienced institutional barriers such as devaluation of experience or expertise. *Individual facilitators* were subdivided and included writing and synthesis as technical skills, networking and collaborating as interpersonal skills, and accountability, leadership, time management, and resilience/grit as personal skills. *Institutional facilitators* included access to mentoring, professional development opportunities, and workload assigned to research. Advocacy for diversity and cultural humility were included as unique interpersonal and institutional facilitators for UMF. Several overlapping and unique barriers and facilitators to mentoring for research success for NI, ESI and UMF in the health-related disciplines are presented.

## 1. Introduction

Being a faculty member in higher education is a rewarding career path that typically involves sharing one’s passion about one’s discipline with hundreds of students, collaborating with faculty colleagues, making noteworthy research discoveries, and traveling around the world to intellectually stimulating places to promote one’s ideas. However, compared to 20 years ago, life as a faculty member has become more challenging. The environment is dynamic, and it includes the corporatization of higher education [1], changing student demographics which may lead to a decline in students pursuing higher education [2], increasing college costs and declining government support [3], COVID-19-related changes affecting teaching and research practices [4], and escalating workload demands brought about by increasing teaching and clinical loads, coupled with intensifying expectations for research performance [5]. Research success is undoubtedly one of the most important components to success in the academy, particularly for those pursuing tenure-track careers.

Being a faculty member in higher education is a rewarding career path that typically involves sharing one’s passion about one’s discipline with hundreds of students, collaborating with faculty colleagues, making noteworthy research discoveries, and traveling around the world to intellectually stimulating places to promote one’s ideas. However, compared to 20 years ago, life as a faculty member has become more challenging. The environment is dynamic, and it includes the corporatization of higher education [1], changing student demographics which may lead to a decline in students pursuing higher education [2], increasing college costs and declining government support [3], COVID-19-related changes affecting teaching and research practices [4], and escalating workload demands brought about by increasing teaching and clinical loads, coupled with intensifying expectations for research performance [5]. Research success is undoubtedly one of the most important components to success in the academy, particularly for those pursuing tenure-track careers.

Faculty in the U.S. are categorized by academic rank, which typically corresponds to their level of experience, and whether or not they are in pursuit of tenure. According to the American Association of University Professors (AAUP), tenure is “an indefinite appointment that can be terminated only for cause or under extraordinary circumstances such as financial exigency and program discontinuation;” tenure was originally conceived to safeguard academic freedom, and to protect faculty who study controversial topics [6].

Tenured and tenure-track faculty in the U.S. are evaluated by several university committees using established criteria, over a longitudinal evaluation period (6–7 years). Evaluation criteria for tenured and tenure-track faculty are mainly related to research accomplishments (e.g., publications, grants, and keynote presentations), but also include teaching and service contributions. Individuals who are tenured are typically Associate or Full Professors (with at least 6 years of experience as professors), and tenure-track faculty (with less than 6 years of experience) are assigned the Assistant Professor rank. Non-tenure-track faculty include lecturers, clinical, or teaching-oriented faculty whose longevity and promotion in the academy depends on their ability to deliver high-quality instruction to students.

One of the most critical times of an academic career is when faculty first accept an academic position. New faculty find that their most dramatic adjustments occur within their first year, and they often struggle to learn teaching, research, and service expectations, department culture and norms, and the political nature of the academy [5]. A key element in obtaining tenure is the expectation to secure external funding, a goal that is also increasingly difficult to attain. A study that examined changes in NIH funding over time (2009–2016) for R01 and research project grants concluded that funding increased 2–5% per year, but that the beneficiaries of this growth were experienced investigators—not new investigators (NI) or early-stage investigators (ESI) [7].

A high percentage of new investigators (NI) and early-stage investigators (ESI) are racial and ethnic minorities or underrepresented minority faculty (UMF) [7,8], which includes American Indian/Alaska Native, Hispanic/LatinX, Black/African American, or Native Hawaiian/Pacific Islander individuals. UMF are not represented in university settings at the same proportion as they are in the general population—especially in academic medical schools [9]. Although the percentage of UMF has increased some over the last several years, the percentage of UMF obtaining tenure and promotion to Full Professor has not increased proportionate to their employment [10].

In addition to ethnically diverse faculty, other persons who are underrepresented in higher education include individuals with disabilities, who were homeless, first-generation college graduates, and/or who grew up in foster care, received free or reduced lunches or Special Supplemental Nutrition for Women, Infants or Children (WIC) or obtained Pell grant assistance [11]. While research exists that examines the impact of mentoring *students* who are disabled [12] or from low SES backgrounds [13], there is a dearth of literature that examines mentoring *faculty* from these backgrounds to enhance research. Given the lack of research on mentoring faculty from these important but understudied categories of underrepresented faculty, this paper will focus on NI, ESI and ethnic minority faculty.

The other complicating factor in the health-related disciplines is that faculty are increasingly difficult to hire [14]. A recent article summarizing nursing graduate opportunities, which are not unlike other health-related disciplines, noted that nursing faculty are becoming harder to find and recruit as these individuals can make higher than average salaries in the private sector [15]. If they do enter higher education, they may leave the field earlier than desired due to campus climate challenges (e.g., lack of opportunity, lack of value or recognition, or ambiguity regarding institutional expectations for academic success) and/or competing personal responsibilities (e.g., family caregiving and work–life balance) [16]. The dynamic and ever-changing milieu within higher education, combined with heavy workloads, can lead to burnout, which is highest among women and underrepresented minorities [17].

One of the most important strategies for helping NI, ESI and UMF succeed is mentoring. Mentoring in the academy is broadly defined as an experienced professor or professors (mentor(s)) supporting a new professor (mentee) in career-related opportunities, challenges and psychosocial areas [18,19]. Contemporary mentoring takes on many different formats including dyadic, peer, or group, and it can occur face to face or via teleconferencing [12]. The numerous career benefits of mentoring have been summarized in literature reviews [20,21] and meta-analyses [22,23,24], and benefits have been examined across disciplines and academic roles such as leadership [25,26], teaching [27] and research [20,21]. Scholars have concluded that effective mentoring provides direction and empowers self-direction [28]. Numerous benefits to mentoring have been cited in the literature including better attitudes, more satisfaction with the work environment, a greater tendency to hold a senior position, and more career success [22]. The impact of mentoring for academic faculty is so strong, that the American Psychological Association [18] wrote that faculty who are mentored typically have more prolific records of publications and grants—and thus earn stronger performance evaluations, higher salaries, and their careers progress faster.

In addition to literature reviews that examined the *overall benefits* of mentoring, other literature reviews in the health disciplines have examined the benefits of mentoring for improved clinical practice [29,30,31,32], and for expanding the research capacity of clinical faculty in the health sciences [33]. Buddeberg-Fisher and colleagues [34] and Byrne and Keefe [35] conducted older narrative literature reviews on expanding research capacity of students and faculty in academic medicine and nursing, respectively. Nowell and colleagues [21] conducted a mixed-methods systematic literature review on outcomes of mentoring in nursing and concluded that mentoring had a helpful impact on behavioral, career, attitudinal, relational and motivation outcomes. One emerging review area is related to expanding global research capacity of students and faculty to improve research and ultimately enhance medical care in developing countries [36].

McRae and Zimmerman [20] conducted a systematic literature review of 34 mentoring programs, with a goal to identify outcomes and components of mentoring programs within the health sciences. Their review focused on programs in medicine, nursing and pharmacy, and it included recommendations for growing research, along with improving teaching and service in academic medicine. Factors present in successful programs included identifying goals at program outset, having regular meetings (monthly or more frequently), providing a formal curriculum, using several methods of mentoring, and providing incentives for mentors and mentees. The most frequently mentioned barriers to program success were finding time to meet and providing training for mentors. They concluded that most programs reported descriptive results, and assessments were conducted using locally designed/context specific measures that were not standardized, and in most cases, not checked for reliability and validity. While this was a comprehensive review, it included mentoring for success in teaching and service in addition to research, and it did not compare mentoring strategies for NI, ESI and UMF.

Chua and colleagues [37] conducted a recent scoping review (2000–2019) of 71 articles about mentoring programs in medicine and surgery. They described three categories important to mentoring novice faculty in clinical, educational, and research settings: the host organization (e.g., academic institution), mentoring stages, and evaluation processes. While this was a comprehensive and valuable review, the majority of studies in this review focused on enhancing clinical skills (68%) rather than research skills.

Confirming the disciplines included in the aforementioned literature reviews, a recent network analysis of mentoring literature concluded that five disciplines emerged as leaders in publishing articles about mentoring: academic medicine (e.g., with 29.2% of studies from family medicine, internal medicine, pediatrics, gerontology, geriatrics, and psychiatry), industrial and organizational psychology (representing 28.7% of studies), education (15.7%), nursing (9.9%) and psychology (8.7%); industrial and organizational psychology and academic medicine have had the most substantial contributions to the literature, as indicated by citations and centrality to the literature [38].

Despite the existing literature on mentoring, little is known about mentoring specifically to advance research in the health-related disciplines, particularly beyond the disciplines of academic medicine and nursing. Most previous reviews are older and have not focused exclusively on mentoring for research development. In addition, an integrative literature review strategy has not been used—which includes additional types of information beyond research articles. A final gap in the existing literature reviews is that to date, no one has attempted to tease out similar and unique barriers and facilitators to research development in NI, ESI and UMF. Given the well-established benefits of mentoring, the ever-changing climate of higher education, the need to review updated contemporary literature, and the lack of an integrative review comparing and synthesizing the literature on research mentoring for NI, ESI and UMF in health-related disciplines, the purpose of this paper was to conduct an integrative literature review to examine the barriers and facilitators to mentoring in health-related research for new and early-stage investigators and underrepresented minority faculty.

## 2. Materials and Methods

Whittemore and Knafl [39] described a framework for conducting integrative literature reviews. This type of review is unique in that it combines diverse methodologies, includes a variety of types of literature, and is specifically designed to contribute to practice, policy, and theory. We felt that examining a more comprehensive variety of literature would enable us to more fully examine effective mentoring strategies and determine whether mentoring for NI and ESI differs from UMF. Our integrative review framework consisted of five stages: (a) developing the research question, (b) searching the literature, (c) evaluating data, (d) analyzing data, and (e) presenting findings.

### 2.1. Research Question

Our research question was: what are the barriers and facilitators to mentoring designed to build research capacity in health-related faculty identified as new investigators, early-stage investigators, or underrepresented minority faculty?

### 2.2. Faculty Categories

In order to fully examine and answer our research question, we felt it was important to clearly define the three (3) faculty categories covered in this review: new investigators (NI), early-stage investigator (ESI), and underrepresented minority faculty (UMF).

**New Investigator (NI).** A NI, according to the NIH, is “an investigator who has not previously competed successfully for substantial, independent funding from NIH.” (See: early-stage investigator status from https://grants.nih.gov/grants/esi-status.pdf.) Institutes and Centers within the NIH fund NIs according to programmatic and strategic priorities.

**Early-Stage Investigator (ESI).** The NIH defines an ESI as “a Program Director (PD) or Principal Investigator (PI) who has completed his/her terminal research degree or end of post-graduate clinical training, whichever date is later, within the past 10 years, and who has not previously competed successfully as PD/PI for a substantial NIH independent research award.” (See: early-stage investigator status from https://grants.nih.gov/grants/esi-status.pdf.) Being categorized as an ESI with a fundable score may be advantageous for receiving grants.

The National Institutes of Health (NIH) developed NI and ESI categories of investigators to promote the “growth, stability and diversity of the biomedical research workforce.” (See early-stage investigator status from https://grants.nih.gov/grants/esi-status.pdf). From an NIH perspective, some of the benefits of NI and ESI status are that (a) the review process focuses more on the approach than the track record, and less preliminary data are required; (b) the NIH has a program for rapid turnaround for NI and ESI applications, which gives them the opportunity to revise and resubmit their applications more quickly (and may be more appropriate for applications with minor issues, but less appropriate for those seeking more thorough feedback); and (c) some grants with ESIs as the Principal Investigator may have more generous pay lines than for PIs with more experience (https://www.niddk.nih.gov/research-funding/process/apply/new-early-stage-investigators).

**Underrepresented Minority Faculty (UMF).** Hassouneh and colleagues [40] clarify an important distinction between faculty of color and UMF. Faculty of color include Asians who are minorities in the U.S., but not in health-related fields, notably medicine. UMF include Black or African American, Hispanic or LatinX, American Indians, Alaska Natives, Native Hawaiians or other Pacific Islanders. Women are underrepresented in certain disciplines, notably academic medicine [41] and STEM fields [42], and sexual minority researchers are underrepresented in scientific leadership positions—even in areas where diseases disproportionately affect them (e.g., HIV-AIDS) [43].

### 2.3. Literature Search

#### 2.3.1. Search Strategy

In Fall 2020, a rigorous literature search was conducted to identify papers relevant to mentoring NI, ESI, and UMF in health-related disciplines. Databases searched included PsychINFO, CINAHL and PubMed. Boolean connectors AND/OR were used to combine search terms including mentor *, research, health, new investigator *, early stage investigator *, minority faculty, and underrepresented faculty. An advanced search process was utilized to limit publications to peer-reviewed journal articles in English, published between 2010 and 2020. After the aforementioned search, to generate additional papers, reference lists were searched and PubMed links were followed to “Similar Articles.”

#### 2.3.2. Inclusion and Exclusion Criteria

Included in this study were peer-reviewed journal articles published in English from January 2010 to October 2020 related to mentoring to increase research productivity in health-related faculty from the three identified faculty categories. We selected this 11-year period of time to: (a) capture how mentoring has evolved due to recent changes in higher education (e.g., high turnover and shortages in health-related faculty, and increased use of different styles of mentoring), (b) ensure that the most current literature was included and synthesized, and (c) capture relevant data from publications featuring recent federally funded projects designed to enhance mentoring of NI, ESI and UMF. We decided to limit publications to those describing research development in the United States given that our federal research funding structure and processes are unique. In addition to published data-based research studies and reviews, essays, editorials, and presentations converted to manuscripts were included. Non-published work, including theses and dissertations, and conference proceedings were excluded.

## 3. Results

### 3.1. Summary of Search Results

**Figure 1:**
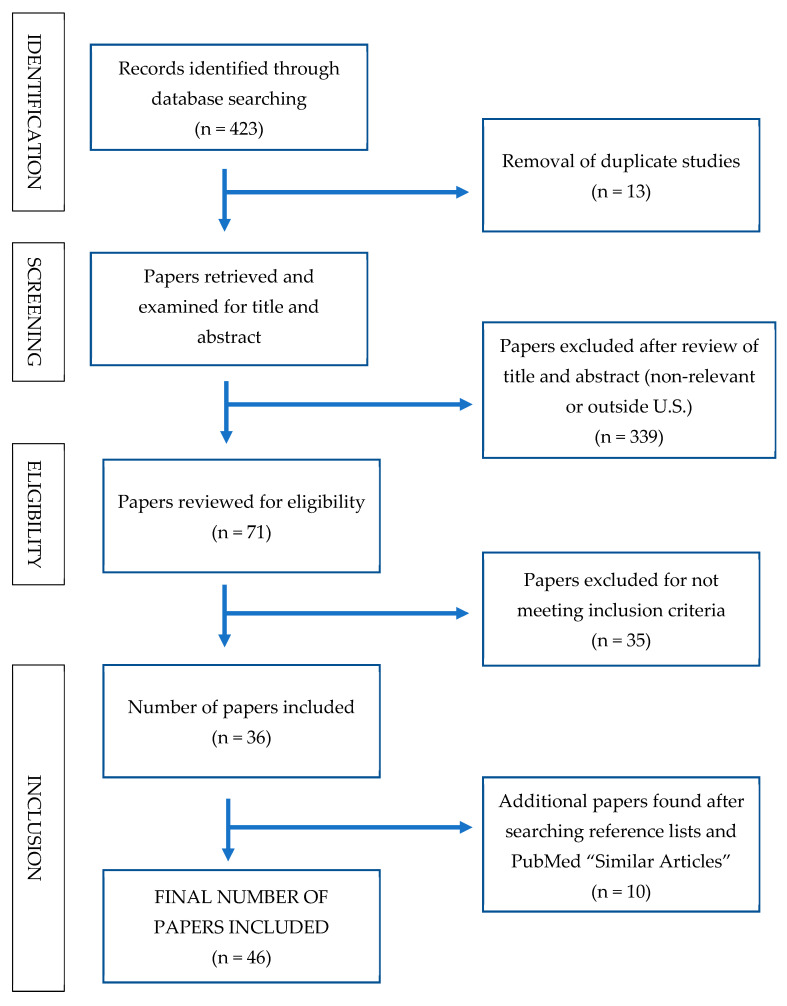
Search strategy and decision trail for selecting included studies.

The database searches generated 423 records. Once duplicates were removed, 410 studies remained. Two authors independently screened the titles and abstracts of papers using the inclusion and exclusion criteria and 339 papers were removed, leaving 71 papers to be reviewed for eligibility. Of the 71 studies reviewed, 35 did not meet inclusion criteria leaving 36 papers. From the remaining 36 papers, references lists were scanned for additional studies, and “Similar Articles” were searched in PubMed. One (1) NIH workshop on mentoring was found. As a result of these expanded search strategies, 10 additional resources were added, resulting in a total of 46 papers. A summary of the decision trail used to locate and select studies for this paper is provided in Figure 1.

### 3.2. Characteristics of Included Studies

Table 1 provides an overall summary table for the 46 papers reviewed, with first authors listed in alphabetical order (identified by reference number), followed by publication year, study design, purpose, methods, participants, additional pertinent results, barriers and facilitators of research (with a focus on mentoring), limitations, and recommended directions for future research.

After completing this review, several important trends in the literature were noted. The health-related discipline most frequently represented in this search was academic medicine (including a variety of specialized medical fields). There were a handful of studies examining mentoring in faculty from behavioral and mental health, nursing, occupational therapy, physical therapy, and public health. The academic content areas in which scholars were most frequently mentored included unspecified or general health (*n* = 21), HIV/AIDS (*n* = 5), and health equity or health disparities (*n* = 4); topics studied less frequently included aging, behavioral or mental health, cancer, obesity and sleep.

Mentoring of faculty in medical and health-related academic units occurred in a variety of formats, including in dyads or constellations, or with peers or groups, and either face to face or via distance (i.e., telementoring). Most papers recommended having multiple mentors (vs. the dyadic approach), and many advocated for assessing baseline research skills and re-evaluating progress regularly [47,56,68,70,84,87].

More than half of the studies (*n* = 27) examined enhancing research capacity in UMF, and a variety of research designs (e.g., descriptive, longitudinal, qualitative, quantitative, mixed methods) were utilized to explore ways in which to enhance research productivity. Although we did not include a search term for “women” as an underrepresented subgroup, several of the studies included a majority of women [49,51,53,59,60,61,63,64,65,81,83,87]. Two of the studies we included exclusively surveyed women [52,84]. We included studies that included a majority of women or reviews focused exclusively on women as they are considered underrepresented in the male-dominated field of academic medicine. Unique aspects of mentoring women were mentioned when they occurred. Just over one-third of the papers (*n* = 18) examined research facilitators in NI and ESI, without significant representation of UMF.

The types of institutions in which the participants were employed varied. Most faculty were from a mix of academic institutions, and results were summarized without disaggregating information about their academic institution. Five studies examined the perspective of NI and ESI faculty in research-oriented institutions, with some exclusively surveying UMF [57,88], and some including a percentage of UMF (10–62%), but not disaggregating findings by race/ethnicity or gender [48,59,83]. Two studies examined research success from the perspective of faculty employed at a minority serving institution (MSI) [44,87]. Three studies described advancing research capacity through partnerships between research-oriented (RO) institutions and MSIs [45,49,71].

In the next sections, we summarize barriers and facilitators of developing research capacity in NI, ESI and UMF in health-related fields. Barriers were both individual (Section 3.3.1.) and institutional (Section 3.3.2.). Individual facilitators were subdivided into technical skills (Section 3.4.1.), interpersonal skills (Section 3.4.2.), and personal skills (Section 3.4.3.), and institutional facilitators were combined and summarized (Section 3.4.4.).

### 3.3. Barriers to Developing Research Capacity

In an effort to identify overlapping and unique barriers to developing research capacity in NI, ESI, and UMF, a concept matrix mapping exercise was conducted. Barriers were divided into individual and institutional factors as Manson [67] noted the importance of considering not only individual constructs, but also environmental (i.e., institutional) constructs.

#### 3.3.1. Individual Barriers to Research Success

Table 2 contains a summary of individual barriers to research success. Individual barriers that appeared in all three (3) faculty categories were a lack of time (*n* = 5) [52,53,57,66,69] and finding work–life balance (*n* = 4) [58,69,76,83].

The most frequently occurring individual barriers for UMF were related to bias and discrimination (*n* = 5) [44,45,60,65,77] and isolation (*n* = 5) [45,60,65,86,88]. Not included in the summary table, yet still worth mentioning as a barrier for UMF (both ethnic minorities and women), is the barrier of differential power dynamics (*n* = 3), which occurs if the mentor–mentee relationship is hierarchical [52], if the mentor does not value the skill set or research area of the mentee [65], or if institutional and mentor values differ from the mentee [88]. Two studies mentioned financial barriers to success including a high cost of living combined with student loan debt, and childcare needs [76,86].

#### 3.3.2. Institutional Barriers to Research Success

Table 3 contains a summary of institutional barriers to research success in NI, ESI or UMF. Institutional barriers were more frequently mentioned than individual barriers. The most frequently occurring institutional barrier for NI, ESI and UMF was a lack of mentors (*n* = 12) [45,46,52,55,63,64,66,67,77,86,88]. Especially lacking were mentors who matched the area of study, expertise, diversity, or research deficits of the mentee. Lack of access to resources was the second most frequently mentioned institutional barrier for all faculty categories (*n* = 9) [44,45,46,48,55,64,65,67,77]. Resources mentioned include research help, teaching buyout, and networks. The third most frequently mentioned institutional barrier for NI, ESI and UMF was a heavy teaching and service load (*n* = 7) [44,45,46,55,64,76].

UMF were significantly impacted by implicit or explicit bias against or devaluation of an academic degree, experience or expertise (*n* = 6) [44,45,57,65,77,88]. Not included in Table 3, but worth mentioning as a barrier for UMF, was a lack of long-term or succession programming for their mentoring programs [16,77,86], and not knowing institution-specific factors that influence success (e.g., promotion and tenure guidelines) [77,79].

### 3.4. Facilitators for Developing Research Capacity

Knowing barriers to research success is helpful, but it is also important to know facilitators in order to overcome barriers and develop corrective strategies. To identify overlapping and unique facilitators for developing research capacity in NI, ESI and UMF, additional concept matrix mapping exercises were conducted. Individual facilitators, which were mentioned more frequently than individual barriers, were further subdivided into technical, interpersonal, and personal skills. Technical facilitators are academic skills (e.g., writing, synthesis, presentations, content knowledge) that early career and UMF faculty have partially developed during their education, but more development is needed to continue to advance a career. Interpersonal skills are those related to the ability to effectively interact and connect with others (e.g., networking, collaborating). Personal skills are traits within an individual, related to success in research, that can be developed with practice and experience (e.g., accountability, leadership, time management, resilience and grit). Institutional facilitators are factors within an institution where a faculty member is employed that boost research success (e.g., access to mentoring, professional development opportunities, workload assigned to research).

#### 3.4.1. Technical Skills That Facilitate Research Capacity

Table 4 contains a summary of technical facilitators that can enhance research productivity for NI, ESI and UMF. The most frequently mentioned technical skills for NI, ESI and UMF, related to research success, were writing manuscripts and grants (*n* = 17), followed by analytical skills (synthesis and statistics, *n* = 4). Analytical skills are vitally important for writing manuscripts and grants, but they are rarely separated from writing in the mentoring literature reviewed. Presentation skills, related to public speaking and thinking on your feet, were also mentioned (*n* = 4) [48,59,65,79]. Two technical skills mentioned less frequently by NI, ESI and UMF, but worth including as facilitators due to their impact on research success, are knowledge about responsible conduct of research [62,72,73] and content knowledge [54,72]. There were no technical facilitators to research success unique to any faculty category—all were consistently mentioned for NI, ESI and UMF.

#### 3.4.2. Interpersonal Skills That Facilitate Research Success

Table 5 summarizes interpersonal skills that are important for growing research capacity. The most frequently mentioned areas in this category that contribute to research success for NI, ESI and UMF are finding productive collaborators (*n* = 33) and networking (*n* = 28). Also important are managing data, projects and teams (*n* = 7) [47,48,62,75,79,87,88], and learning organizational dynamics and navigating political traps (*n* = 6) [46,63,65,73,76,88]. An interpersonal skill not included in the table, but mentioned in some papers is responding to feedback (*n* = 5) [43,46,47,78,88]. Compared to being defensive about feedback, responding appropriately can facilitate continued corrective mentoring, and result in more published papers and funded grants.

One important interpersonal skill mentioned in a large number of the papers on mentoring research success in UMF was advocacy for diversity and cultural humility [55,57,60,62,65,70,71,72,77,80,87,88]. Berget and colleagues [45] described “survival skills workshops” that included creating a sense of community within a research discipline; effectively articulating benefits of CBPR and community partnerships to peers and administrators; and appropriately saying no if asked to participate in too many minority-related service assignments. Having frank discussions about family–work–life balance, time management, and building coalitions of internal and external supporters were also deemed important. Flores et al. [62] described essential strategies for research success for UMF, which included advocating for diversity and cultural humility by debriefing often with colleagues, handling implicit bias professionally, and responding to bias immediately (vs. cumulatively). Both Flores et al. [62] and Jean-Louis et al. [65] mentioned the importance of failing productively and having a safe space to fail.

#### 3.4.3. Personal Skills That Facilitate Research Success

Personal skills needed for success in a research career are summarized in Table 6. The most frequently mentioned personal skill for NI, ESI and UMF is accountability (*n* = 17), followed by career planning (*n* = 14). Mentioned less frequently is leadership [47,59,62], resilience and grit [47,62], and ethical behavior (e.g., treatment of human subjects, data collection and management, citations, authorship) [47,72]. There were no personal facilitators unique to research success—all were consistently mentioned for NI, ESI and UMF.

#### 3.4.4. Institutional Facilitators of Research Success

Table 7 presents institutional facilitators that are essential for research success. The institutional facilitator mentioned most frequently for NI, ESI and UMF was access to expertise and mentoring (*n* = 45), followed by professional development opportunities (*n* = 38), science culture (*n* = 17), workload assigned to research (*n* = 16), and funding (*n* = 14). Five papers recommended pre-screening research skills to determine the best mentors and mentoring strategies for each mentee [47,56,68,70,84], and one paper recommended this strategy for UMF [38]. Knowing the promotion and tenure standards, which was mentioned, but not included in the table, was considered an important institutional facilitator by Blanchard et al. [43], Feldman et al. [59], Flores et al. [60], and Martina et al. [68].

Sixteen papers advocated for developing mentoring strategies leading to cultural humility and a culturally responsive institution, and of these, 13 included scholars of color in their sample [44,46,53,55,57,58,60,61,62,65,71,77,88]—one was a review about women in higher education [52], and two were focused on NI and ESI [67,70].

## 4. Discussion

The purpose of this paper was to conduct an integrative literature review to examine the barriers and facilitators to mentoring in health-related research, particularly for NI, ESI or UMF. One important general finding of this integrative literature review was that health-related research should continue to expand the disciplines and content areas in which mentoring studies are conducted. We reported that the majority of mentoring studies included in this review focused on academic medicine, which is similar to findings reported by Lefebvre, Bloom, and Loughead [38]. Although we did not search for any health-related disciplines by name, we did use “health” as a search term, which garnered studies from other disciplines. Given differences in workloads, accreditation requirements, and institutions, it is important to continue to study mentoring in other health-related academic disciplines, across academic ranks (including mid- and late-career faculty), across intersectionality (e.g., minority women), and across various types of academic institutions (e.g., minority serving institution, research-oriented institution or emerging research institution).

A second general finding was that mentoring occurs in a variety of formats, and researchers increasingly recommend pre-assessing research skills so that mentoring teams can be formed, which can help NI, ESI and UMF receive support in as many desired areas as needed. We noted that numerous types of mentor–mentee matching strategies were used including dyads, peers, groups, constellations or groups, and telementoring. Prior researchers [27,89,90] have categorized formal mentoring similarly.

We conclude that both formal and informal mentoring are important for research development. Formal mentoring systems match mentors and mentees based on personality, culture, goals and expectations [90], and they typically have planned, regular activities that consider developmental stages of the mentees [91]. Informal mentoring, which is unstructured, consists of mentees discussing career strategies and aspirations, along with professional, personal and psychosocial issues, with mentors [92]. Huggett and colleagues [93] concluded that informal mentoring is important for career satisfaction and formal mentoring is important for enhancing academic productivity. Some programs recommend discipline- or department-specific mentors [94,95], while others advocate for cross-departmental mentoring, which helps with meeting a variety of research development needs [53,90] and with confidentiality [96]. Brown, Daly and Leong [95] advocated for a “developmental model of mentoring,” whereby different mentoring strategies are used for each level of researcher in psychology (e.g., undergraduate and graduate student, post-doc, and junior faculty) and for women and ethnically diverse researchers.

### 4.1. Individual and Institutional Barriers to Research Mentoring

The most prominent individual barriers to mentoring for research success, which were mentioned for NI, ESI and UMF, were a lack of time, and finding work–life balance. Due to the increasing expectation that faculty members do more with less, it is unlikely that time will be removed as a barrier to research mentoring. The goal then, should be to achieve maximal benefits in minimal time. This can be done by pre-assessing NI, ESI and UMF for research readiness, focusing heavily on research development areas identified in the needs assessment, and sharing the mentoring load among multiple mentors. Given that potential research mentors are also coping with increased demands, providing training and incentives (e.g., workload credit, authorship, or renumeration) for mentors may more effectively incentivize them to participate.

It is disconcerting that unique individual and institutional barriers are still present for UMF. The most prominent barriers reported by individual UMF were related to bias and discrimination. Bias can include negative experiences with hiring, promotion and tenure evaluations, or manuscript/grant reviews, or it can involve perceived decreased value of degrees from certain institutions. Individual and institutional barriers are intimately interrelated for UMF, such that when they encounter institutional barriers to research success, it exacerbates individual barriers such as perceived racism. For example, when UMF are hired into predominately white institutions of higher education, they report that they experience overt and covert racism, their work is marginalized, and their contributions are undervalued; in addition, UMF are often asked to participate in activities that will not advance their careers (i.e., serve as minority representatives on committees), prompting some UMF to report feeling like “institutional mascots” [97]. As a result of these experiences, some UMF struggle with a sense of isolation (i.e., “being the only one”) or “imposter syndrome,” meaning that they feel that they do not belong in a specific setting [88]. Others report “unpleasant peer interactions” that isolate them academically and socially, and may prevent them from “accumulating social capital” [88]. A lack of mentoring has left many UMF feeling like they did not have the information and guidance they needed to succeed in networking (internally and externally) and meeting tenure-and-promotion expectations [88,90,98]. When these issues are combined, many UMF experience work stress in the academy, which in turn, can contribute to stress-related health problems [88,97].

Another individual barrier for UMF is coping with differential mentoring power dynamics. The concept of “differential power dynamics,” which appeared in papers summarizing concerns of minorities [65,88] and women [52], refers to the use of a hierarchical model of mentoring, whereby the mentor holds the rank and power, and the mentee is required to follow the direction of the mentor—whether or not it is desired by the mentee. Ideally, mentoring relationships are bi-directional, whereby both the mentor and mentee receive benefits. Mentoring opportunities and settings can be structured in ways that minimize power differentials and encourage the exchange of ideas, mutual responsibility, and flexible options for the mentor and mentee.

The most frequently mentioned institutional barrier to research mentoring that overlapped across NI, ESI and UMF was a lack of mentors. Mentors may be less willing to participate if they are not receiving workload credit for mentoring or if the experience is not mutually beneficial. Additionally, prior research has reported that some institutions (e.g., predominately white institutions) lack diverse faculty who can mentor [88], and some academic disciplines (e.g., medicine) lack women and diverse faculty who can mentor [41]. Sometimes, mentors are assigned without knowledge of an academic discipline or method of collecting data. Pre-assessing mentoring needs and then assigning mentoring teams may help offset this challenge. Mentoring needs assessments can be structured in a way that focuses on pairing mentees with mentors based on faculty strengths, previous accomplishments, and interests in ways that maximize information sharing to help with the formation of mentoring teams.

Another barrier mentioned was a lack of resources, such as access to journals, insufficient funds for research costs (pilot data collection or research help), inadequate buyout from teaching, or reduced access to research networks. Related to a lack of resources is a heavy teaching and service load, especially for those working at minority serving institutions [44], juggling clinical responsibilities in addition to teaching and research [76], or serving on various committees to represent their minority group interests [45].

### 4.2. Individual (Technical, Interpersonal, and Personal) Facilitators of Research Mentoring

Facilitators of research mentoring, which overlapped in NI, ESI and UMF at the individual level, were technical skills such as help with writing manuscripts and grants, statistics and synthesis, or oral presentations, interpersonal skills, such as help with finding collaborators, networking, managing teams, and navigating organizational dynamics, and personal skills such as holding mentor and mentee accountable and helping with career planning. Not surprisingly, these individual mentoring skills are offered by most mentoring workshops and programs that seek to help investigators at the individual level.

### 4.3. Institutional Facilitators of Mentoring for Research Success

The most frequently mentioned institutional facilitators of research success for all faculty categories included access to expertise and mentoring, professional development opportunities, science culture, workload assigned to research, and funding. Most of these needs appeared first as barriers, and now as facilitators.

Advocacy for diversity and cultural humility were interpersonal research facilitators specific to UMF. Advocating for diversity can include insisting that applicant pools and subjects for research studies are diverse, recruiting diverse individuals to serve on faculty and in other important roles, and supporting diverse individuals who speak for specific types of candidates and research agendas, or speak out against the minority tax (e.g., being asked to do twice as much service to “represent” an underrepresented group) [99]. Cultural competence refers to “mastering a theoretically finite body of knowledge,” whereas the newer term, cultural humility refers to “a lifelong commitment to self-evaluation and self-critique, redressing power imbalances that occur throughout the academy, and developing mutually beneficial and non-paternalistic clinical and advocacy partnerships with communities” [99]. Interpersonal diversity-oriented strategies can be reinforced at the institutional level by training mentors to utilize culturally responsive mentoring strategies and advocate for a culturally responsive institutional climate. Advocacy for diversity and cultural humility can go a long way toward addressing both the individual and institutional barriers that UMF feel related to bias and discrimination. Educating all faculty about implicit bias and cultural humility, and discussing the importance of advocating for diversity and understanding ethnically relevant research methodology could prevent UMF from feeling like “tokens” or “ethnic specialists.” These activities may also lessen some of the isolation typically felt by UMF because the responsibility to discuss equity, inclusion and diversity will fall upon more faculty colleagues—not just the UMF employed at a university.

Universities should be a place where culture is discussed, conceptualized, and valued [20], and where individuals authentically address bias, stereotype threats, and cultural ignorance [59]. Emphasizing the importance of cultural humility requires individuals to self-evaluate and self-critique, advocate against power imbalances, and develop partnerships with people and groups who advocate for others who are different from themselves [100].

Interestingly, five of the six studies that recommended pre-assessing faculty for research readiness were specific to NI and ESI; only one mentioned pre-assessing the research readiness of UMF. This presents an opportunity for institutions of higher education to develop UMF-specific mentoring programs that conduct needs assessments for research development, match multiple mentors with a mentee, regularly re-assess, and measure outcomes.

### 4.4. Limitations

Despite the plethora of findings reported, this study was not without limitations. Studies included were relevant to NI, ESI and UMF, limited to an 11-year time period, written in the English language, and all were from the United States from health-related disciplines. While some of our results may be generalizable to other disciplines or other countries, caution is urged as this paper is focused on faculty from the health disciplines, during a specific time period of their career, within NI, ESI and UMF utilizing data from institutions of higher education in the United States. This review did not include theses or dissertations or studies examining research mentoring in scholars with disabilities, or those who come low SES backgrounds. We were not able to locate specific literature that has examined growing research productivity in those categories of underrepresented faculty. Additionally, we were not able to distinguish between mentoring needs of subpopulations of UMF. For example, needs of an African American scholar may differ from those of a Hispanic or Native American scholar. The literature did not delve more deeply into this very important issue, so we were not able to explore this topic. Another limitation is that terms related to mentoring and measures of impact are not standardized across studies, which may conflate findings. The use of an integrative literature review technique is qualitative in nature, and does not include effect sizes or other quantitative data that might inform the reader about the magnitude of effects of certain mentoring behaviors. Finally, although this review was comprehensive, some articles related to this topic may have been unintentionally omitted.

### 4.5. Future Directions

Our integrative review of the literature uncovered several areas that would benefit from future research. First, the mentoring literature could be advanced by comparing mentoring at all career stages (e.g., early, mid and late career) and making recommendations for best practices at each stage [85]. Second, there is a need to expand studies examining mentoring for research development beyond the U.S. Nowell et al. [21] reviewed literature on the outcomes of mentoring nursing faculty from the U.S., Canada, and Australia, and Chua et al. [37] examined three themes (host organization, mentoring stages and evaluation) in mentoring programs from the U.S., the UK, Canada, and other countries. However, even though studies from other countries have been included in previous reviews, a comparison of how research mentoring programs differ across countries has not been conducted. From a critical analysis of worldwide research mentoring programs, policy recommendations can be made to enhance research productivity—either at the individual or institutional level. Relatedly, more research should be conducted to examine the impact of cultural or ethnic background on research development. Specifically, Espino and Zambrana [57] noted that when the research productivity of underrepresented groups has been studied, Native Americans, those who are differently-abled, LGBTQ, or those from disadvantaged backgrounds (e.g., homeless, foster care, first-generation college graduates, or low SES) are rarely included. Further examining how underrepresented groups may differ from one another in mentoring needs should also be considered. Thirdly, little research has been conducted to test the effectiveness of different types of mentoring (e.g., dyads, groups, peers, and telementoring) [89]. Fourth, we need to expand the types of studies conducted beyond descriptive, cross-sectional and qualitative, to include more longitudinal, prospective and experimental/intervention designs [56,82]. Fifth, scholars could expand the insights provided by reporting more comprehensive details on institutional and departmental characteristics and differences between mentees, mentors, and mentoring programs, as well as reporting on intersectional characteristics that define a faculty member and may impact career success (e.g., LGBTQ, differently-abled, military, first-generation college graduates, or low SES backgrounds). Sixth, taken as a whole body of literature, only a handful of studies included and described a theoretical framework [41,44,48,69,77]. Literature reviews by Doyle et al. [54] and Pfund et al. [72] expressed the need for continuing to advance the theoretical framework for mentoring in the academy. As a seventh direction for future research, there is a need to standardize mentoring terminology and evaluation methods [54,72,74,89] and continue to examine the most important variables (e.g., mediators) for enhancing mentoring. For example, Thorpe et al. [83] emphasized that there is a strong relationship between academic self-efficacy (i.e., grant writing self-efficacy) and academic success [47], but additional research is needed. Although two reviews on assessing mentoring success have been published [101,102] it is clear that more research is needed to discern which questionnaires, surveys, interview questions, or other strategies should be used to assess the success of specific parts of mentoring programs (e.g., mentor and mentee success and program success). An eighth recommendation is to examine not only the benefits, barriers and facilitators of mentorship for mentees, but also appropriate content and strategies for training mentors [89], and how that relates to program success. A ninth recommendation for future study is to continue to examine how to pair mentors and mentees for research success, as was discussed in Huggett et al., [93] and Huskins et al. [103]. To date, researchers have recommended mentor–mentee matching based on educational background, professional experience, teaching or research assignments, professional interests, and mentee goals [89]. However, studies have failed to compare different strategies for matching mentees with mentors to determine whether different methods of matching result in different outcomes. Finally, the ultimate study design might examine which types of mentoring (dyads, peers, etc.) and which modes of mentoring (formal, informal), as moderated by gender, ethnicity or intersectionality, and mediated by academic environment and career stage, have the most impact on academic success [72,77].

## 5. Conclusions

In this study, we sought to answer the research question: “What are the barriers and facilitators to mentoring designed to build research capacity in health-related faculty identified as NI, ESI or UMF?” Our study is unique in that it expanded health-related disciplines previously examined beyond nursing and academic medicine using an integrative literature review. It also examined individual and institutional barriers and facilitators to research development that were similar and unique in NI, ESI and UMF. Many individual and institutional research barriers and benefits were consistent across faculty subgroups, but there are some that are institution-based or unique to UMF. Specifically, the most frequently mentioned individual barriers were lack of time and finding work–life balance. UMF expressed concern about the impact of bias, discrimination and isolation on research productivity. Individual facilitators should target the aforementioned barriers, and build skills in writing/synthesis, networking, accountability, leadership, time management and resilience/grit. Institutional barriers include a lack of mentors, access to resources, and a heavy teaching/service load. Institutional facilitators should seek to decrease the aforementioned barriers, and increase access to mentoring, professional development, and workload assigned to teaching. Advocacy for diversity and cultural humility are important career facilitators for all faculty, and offering training at the institutional level establishes the importance of these strategies for career success. Mentoring, if done systematically with a focus on individual and institutional barriers and facilitators, using evidence-based practices, should have a positive effect on the success of NI, ESI and UMF. Stoff [79] purports that increasing diversity in research personnel will lead to decreased health disparities and increased health equity among underserved populations; in addition, diverse teams capitalize on innovation and bring different perspectives, creativity and experiences to address complex scientific problems. The bottom line is that if diverse health-related faculty are successful in research, this should have a positive effect on health behavior and health promotion, and it should lessen the burden of disease in our most vulnerable populations.

## Figures and Tables

**Table 1:** Summary of studies examining barriers and facilitators to research in health-related faculty categorized as NI, ESI, or UMF.

[Reference Number] Author(s) (Year)	Study Design, Purpose, and Methods	Participants (Diversity, Faculty Category) and Any Additional Results	Barriers to Research	Facilitators of Research	OTHER: Limitations, Future Research Directions
[44] Beech et al. (2020)	Design: Descriptive, qualitative Purpose: Examine research challenges of early career faculty from HBCUs (teaching-intensive institutions) Methods: 90 min discussions of barriers to research, overcoming barriers, experiences with research mentors, factors facilitating research	Participants: ESI and research leaders from HBCUs in Mississippi and Baltimore, MD	Barriers: (1) Access to resource; (2) bias; (3) heavy teaching and advising load	Facilitator: (1) Engaging students of color in research—may stimulate future interest in research	Future Directions: These findings were utilized to develop their research training and mentoring program
[45] Berget et al. (2010)	Design: Descriptive, mixed methods, program evaluation Purpose: Evaluate Summer Research Career Development Institute (SRCDI) which teaches emerging minority investigators research skills Methods: Post-program eval. of 2 institutes with questionnaire and oral feedback; second questionnaire 12 months after completing institute	Participants: 55 post-docs or junior faculty researching in health equity from the U Pittsburgh–Jackson State U partnership	Barriers: (1) Treated as “token hires” or “ethnic specialists” (vs. experts); (2) isolation and chilly campus climate; (3) shortage of mentors; (4) spending valuable time on committees; (5) no access to informal networks; (6) majority faculty determine merits of academic endeavors	Facilitators: (1) Survival skills classes; (2) problem-based learning to facilitate research ideas; (3) scientific autobiography from sr. minority faculty; (4) junior faculty panel; (5) writing/publication strategies; (6) network of peers; (7) MOST USEFUL: mock reviews, presentation practice, networking	
[43] Blanchard et al. (2019)	Design: Mixed methods Purpose: Report outcomes of an junior minority faculty mentoring program from 2008 to present Methods: 18-question survey + qualitative interviews	Participants: Junior and senior members of NMRI		Facilitators: (1) Prof. and personal mentors; (2) mock study sections; (3) support for writing NIH grants; (4) P and T feedback; (5) grant/manuscript writing and mgmt.	Future Directions: Study to determine whether annual program feedback is correlated with career advancement
[46] Brewer et al. (2016)	Design: Qualitative Purpose: Examine needs, challenges, contributions, and successes in mentoring for underrepresented early career faculty	Participants: 4 Black early career investigators + 15 underrepresented investigators from the U.S.	Barriers: (1) No mentor network; (2) unstructured mentorship; (3) mentor mismatches; (4) failing to balance service/research opportunities	Facilitators: (1) Mentor–mentee match; (2) network of mentors invested in mentee success; (3) guidance for navigating political landscape; (4) institutional supports	
[47] Buist et al. (2017)	Design: Descriptive Purpose: Describe the goals and components of the CRN program. Methods: 26 month program to increase capacity for cancer research; scholars ID mentoring and workshop needs	Participants: 28 junior investigators from 14 CRN sites or academic centers		Facilitators: (1) Protected research time and buyout; (2) multiple mentors—who receive credit for effort; (3) planned activities; (4) research skill development; (5) multisite collaboration—exposes scholars to more expertise; (6) virtual data warehouse	Limitation: Hard to tease out which components contributed to success since this was an evaluation and not an experimental design
[48] Byington et al. (2016)	Design: Descriptive Purpose: Describe clinical and translational scholars (CATS) mentoring program Methods: 2 years of mentorship for ESI engaged in biomed. rsh. transitioning to PI. Faculty nominated by chairs/deans and receive at least 30% effort for research	Participants: 86 scholars accepted between 2008 and 2015 Results: 46% female, 10% UMF; 92% had extramural funding by program graduation; 99% remained in academic medicine	Barriers: (1) Cost to run the program—BUT based on grants awarded, return on investment of the CATS program is more than 20 to 1	Facilitators: (1) Move away from dyadic mentoring toward mentoring networks	Limitations: Reporting data for single-center study and short follow up for each scholar
[49] Campbell et al. (2013)	Design: Descriptive Purpose: Summarize outcomes of career training and research practices by faculty from teaching-intensive, minority serving institutions through a visiting professorship (VP) program Methods: Faculty receive 8–10 weeks training in the labs of host scientists at research-intensive institutions	Participants: 32 participants, 60% female, majority Black and Hispanic, and from 1997 to 2008 93% URM Results: More independently productive than matched peers		Facilitators: (1) Modest annual financial investment (~$6000/person) and workshop offered; (2) commitment of host scientists; (3) willingness of home institution to allow program; (4) large professional associations focused on minority success	
[50] Cohen et al. (2012)	Design: Case control Purpose: Identify characteristics of successful mentoring programs Methods: Institution categorized based on # of plenary research presentations at Nat’l convention over 6 years; questionnaires for mentors and mentees	Participants: 159 professors (97 mentors) and research fellows (62 mentees) at Career Stage: mostly male mentors (Sr) and female mentees (ESI)		Facilitators: (1) Protected research time; (2) easy, formal program that connects mentors and mentees with similar research interests; (3) feedback on research; (4) mentee progress reports required (accountability)	Limitation: 46% response rate (although comparable to other published studies)
[51] Comeau et al. (2017)	Design: Mixed methods Purpose: Evaluate the impact of the NIH National Center for Research Resources (NCRR) Mentored Clinical Research Scholar (CRS) Program Award K12 and the ACTSI KL2-Mentored Clinical and Translational Research Scholars program Methods: Mentored clinical research training. *Quantitative*: demographics, publications, grants. *Qualitative*: interviews	Participants: Junior faculty physicians at Emory University interested in clinical/translational science careers Results: 46 ESI have been supported by K12 and KL2 programs (65% women, 22% underrepresented minority; 100% reported they were very satisfied with the program		Facilitators: (1) Mentors = significant role in idea generation, study planning, and design of research studies; and review of grant drafts and manuscripts; (2) mentoring teams were helpful to long-term career success; (3) a funding mechanism; (4) protection of time; (5) quality of training provided	Future Directions: (1) More research methods classes related to diverse populations; (2) more opportunities to learn about software programs; (3) debrief with program administration; (4) add opps. for timely feedback
[52] Cross et al. (2019)	Design: Literature review of papers from 2000 to 2018 (*n* = 27 studies and 8055 women) Purpose: Uncover factors associated with effective mentoring of female health academics, the consequences inadequate mentoring, and gaps in knowledge		Barriers: (1) Personal/relational dynamics (e.g., variable quality of mentors and incongruent assignment of mentors, power dynamics); (2) lack of senior women; (3) time to mentor	Facilitators: (1) Mentor availability and expertise; (2) supportive relationships (mutuality, responsiveness to needs)	
[53] Cruz et al. (2020)	Design: Descriptive Purpose: Share results of the first 3 years of Transdisciplinary Research, Equity and Engagement (TREE) Pilot Program Methods: Pilot funding awarded (up to $50,000) to conduct behavioral health disparity research. PIs develop mentoring plans for a mentor. TREE leadership also mentors pilot PIs individually	Participants: 10 projects awarded: 6 female, 5 Hispanic/Latinx, 1 Native American, 1 sexual minority Results: All PIs engaged with community partners on research and disseminating results	Barriers: (1) Difficult to accomplish work in 1 year time frame; (2) lack of designated time for PIs to devote to pilot projects; (3) difficulty integrating pilot research into other TREE Center activities	Facilitators: (1) Mentors willing to mentor UMF; (2) mentors who engage w/community; (3) dedication of mentor/mentee; (4) network diverse faculty from two campuses; (5) have an academic and community mentor; (6) bimonthly research roundtables	Future Directions: (1) Provide more access to grant and manuscript reviewers who are subject matter experts; (2) connect investigators to community partners; (3) address institutional challenges
[54] Doyle et al. (2019):	Design: Literature review Purpose: Provide an overview of research focused on mentoring practices and related outcomes in OT Methods: 1313 studies found and 20 reviewed	Participants: OT students and professionals including clinicians, educators, and researchers		Facilitators: (1) Create plan, adhere to expectations, meet frequently; (2) use goal setting, problem solving, resource sharing, critical thinking, reflection; (3) provide support (trust, commitment, respect)	Future Directions: (1) Define mentoring and develop std. measures; (2) describe theoretical background; (3) expand beyond XS research
[55] Duncan et al. (2016)	Design: Qualitative Purpose: Identify issues that impact the training and retention of underrepresented individuals in biomedical research Methods: 5 independent focus groups covered 5 topics affecting UMF in biomedical sciences	Participants: ESI invited to workshop Results: Effectiveness of current NHLBI diversity programs = increased independence; early programs important for skill development; need to support key aspects of institution mission	Barriers: (1) Socioeconomic. Challenges (cost of living, high student debt, lack of child care, sacrificing career for family, health insurance, retirement benefits). (2) Non-PI career paths in research (limited opps. for R1s in teaching institution)	Facilitators: (1) Academic community promoting diversity: networking; multitiered mentoring approach; (2) NHLBI diversity programs (mock grant reviews; helpful info from successful scientists); (3) find research “niche” and secure funding	Future Directions: (1) Use online technology to facilitate collaborations; (2) incentives for mentors; (3) ID mentors within NHLBI community; (4) form partnerships with other scientific societies
[56] Efstathiou et al. (2018)	Design: Mixed methods Purpose: Assess short (pre–post) and long-term (7 years post) impact of a formal mentor program on junior faculty satisfaction and productivity in academic medicine Methods: Prospective longitudinal intervention (2009–2016); 3 formal training sessions over 9 months + regular informal meetings	Participants: 23 junior faculty mentees who participated vs. 91 junior faculty controls; from radiation oncology, anesthesia, critical care, pain management		Facilitators: (1) Ask mentees to rank their top 5 areas of professional development need and mentors to rank their top 5 mentoring strengths and pair them; (2) provide training to mentors; (3) provide guidance on how mentee and mentor can collaborate on joint expectations, goals and timelines	Limitations: Small sample size, unmeasured confounders Future Directions: More longitudinal and intervention studies
[57] Espino and Zambrana (2019)	Design: Mixed methods Purpose: Perceptions of 58 URM faculty employed at U.S. research-extensive universities on mentoring Methods: Focus group and individual interviews and survey data from RWJF National Faculty Survey	Participants: 58 UMF (African American, Mexican American, and Puerto Rican at the Asst/Assoc Prof rank) employed at predominately white U.S. research-extensive universities; mixed research disciplines including health	Barriers: (1) Working more hours/week; (2) older age; (3) organizational value of independence	Facilitators: (1) Formal + informal mentoring; (2) match pairs—including on racial/ethnic concordance; (3) mentoring viewed as a partnership vs. hierarchy; (4) mentors trained; (5) meet regularly; (6) career-related and psychosocial mentoring; (7) strong mentor commitment; (8) mentoring outcomes assessed	Limitations: XS design; voluntary nature of participants = possible selection bias and social desirability of responses; Native Americans not included Future Directions: Explore impact of the different types of mentoring
[58] Felder et al. (2019):	Design: Descriptive, quantitative Purpose: Explore differences in the personal characteristics, mentoring, training, and scholarly productivity of a diverse sample of trainees in the US by NIH underrepresented status Methods: Web-based questionnaire	Participants: Students, post-docs, faculty from 23 NCI/NIH-funded CNPCs Results: Sharing personal or cultural characteristics with CNPC mentor is extremely important to URF	Barriers: (1) UMF were more likely to be first-generation college graduates; (2) UMF with higher satisfaction with work–life balance and current position less likely to have grant funding	Facilitators: (1) 38% of CNPC mentors also first-generation college graduates; (2) having ≥1 mentor was a significant predictor productivity outcomes	Limitations: Cross-sectional analysis. No examination of previous mentoring experiences, only CNPC experiences
[59] Feldman et al. (2010)	Design: Descriptive quantitative Purpose: Determine characteristics associated with having a mentor, the association of mentoring with self-efficacy, and the content of mentor–mentee interactions Methods: 38-item, web-based survey	Participants: Pre-tenure faculty (*n* = 464) in dentistry, medicine, nursing and pharmacy, mostly white (62%) and female (53%)		Facilitators: (1) Finding mentor themselves; (2) research faculty line vs. clinical line; (3) top 10 topics: obtaining funding/grants, writing manuscripts/grants, research design, career planning, Tenure & Promotion expectations, time management, pres., networking	Limitations: Prior programs had small sample sizes, were informally organized, and had difficulty with long-term sustainability
[60] Flores et al. (2016)	Design: Descriptive Purpose: Discuss 6 “hot topics” related to research success in young ethnically diverse investigators Methods: The Research in Academic Pediatrics Initiative on Diversity (RAPID) convention held a “Hot Topic” session covering 6 topics on research success	Participants: 10 young ethnically diverse investigators and 5 senior investigators	Barriers: (1) Racism and discrimination; (2) coping with isolation as a minority faculty member; (3) lack of clarity about T & P requirements	Facilitators: (1) Protected time for research—with concrete steps, timelines, and outcomes; (2) professional and personal mentoring—internal and external to your university; (3) social support; (4) applying for RFAs for NIH grant opportunities for minorities	
[61] Flores et al. (2020)	Design: Descriptive Purpose: Evaluate the RAPID program Methods: Small research grants awarded ($15,000 for 1 year), with mentoring, networking and career development	Participants: 10 scholars (8 women, 6 Latinos, 3 AAs from 8 institutions) from first 4 cohorts		Facilitators: (1) Funding for grants ($15,000/year); (2) mentoring by senior investigators; (3) networking and career development at annual conference; (4) monthly mentoring	Limitations: Evaluation was not conducted as an RCT, small sample size
[62] Flores et al. (2019)	Design: Descriptive Purpose: Provide a guide to academic success for URM young investigators using the 2018 RAPID conference panel discussion Methods: 6 key questions using an expert panel	Participants: Heterogeneous panel of experts representing both genders, multiple races/ethnicities and geographic diversity across the U.S.		Facilitators: (1) Multiple mentors; (2) write prolifically; (3) persistence and fail productively; (4) debrief with colleagues; (5) seek non-traditional funding streams; (6) balance committee work with research; (7) ask for resources (protected research time); (8) handle implicit bias professionally and respond immediately; (9) serve on an NIH study section	
[63] Harawa et al. (2017)	Design: Mixed methods Purpose: Discuss optimal approaches for mentoring programs for URMs in health research careers in the resource Centers for Minority Aging Research (RCMAR) program	Participants: 361 scholars from 12 centers. 66% members of UMF groups, 72% women, 80% remain in academia Results: Centers outlined their approach to selecting, training, and matching mentors, training scholars, evaluation, and addressing issues prevalent among URM scholars	Barriers: (1) Small number of scholars and mentors in each center; (2) worried about lack of anonymity in evaluations that leads to negative consequences (one center replaced evaluation surveys with facilitated, focus group-like discussions among scholars—more informative).	Facilitators: (1) Centers rely on multidisciplinary mentoring teams; (2) formal scholar training provided by all centers; (3) RCMAR scholars later served as RCMAR faculty; (4) mentoring continues between scholar cohorts; (5) regular contact (monthly); (6) accountability = formal agreements between mentors and scholars; (7) attend designated trainings to share work with other RCMAR folks	
[64] Hemming et al. (2019)	Design: Descriptive Purpose: Provide a picture of national applicant pool and test for differences between underrepresented groups (URGs) and well-represented groups (WRGs) and institution type (MSI or minority serving institution vs. other) in variables that might influence an investigator’s success in developing, submitting, and acquiring research grant proposals	Participants: 880 people who submitted online applications to join an NRMN Grantsmanship Program Results: 50% URGs, 65% female. URGs published < articles, spent < time on grants, and research than WRGs; MSI faculty < likely to have collaborators in their research area and < time to conduct research	Barriers: (1) No release time or buy out for research; (2) less expertise relative to obtaining federal funding; (3) UMF less likely to report access to research resources (core facilities to conduct research and reside in a department where majority of faculty had external funding)	Facilitators: (1) Link URGs to networks where they can collaborate and access facilities that advance their work; (2) increase access to professional development programs that prioritize skill development and intentional efforts to embed URGs in pertinent research networks; (3) negotiate release time	
[65] Jean-Louis et al. (2016)	Design: Mixed methods Purpose: Report preliminary results on mentee’s satisfaction with institute components and academic success contrasting two specific outcomes (# of pubs and grant awards) before matriculation, during, and upon program completion Methods: Programmatic activities = Summer I sessions, mid-year meeting, monthly webinars, and Summer II sessions. Quantitative data from web-based evaluation system and qualitative data from interviews/focus groups with 17 mentees	Participants: 29 URM mentees from 15 US institutions selected for participation in NYU PRIDE Institute. 66% female, 79% Black, 17% Hispanic, and 4% White with disability Results: Lectures were effective and interesting. Mentoring important. Overall proposals submitted to the NIH increased during and after completing the institute. Growth in # of mentees submitting grant applications or publication records related to increased academic self-efficacy	Barriers: (1) Challenges experienced by UMF were fundamentally different than non-URM colleagues; (2) being the only URM faculty member = isolated and misunderstood; (3) colleagues do not talk about work in terms of scholarship and service, they talk about it in terms of dollars	Facilitators: (1) PRIDE helped combat feelings of marginalization and provided opportunity to step away from grind and refocus/rekindle desire to conduct research; (2) PRIDE referred to as “safe space to learn and make mistakes”; (3) URM PRIDE staff understood the mentees experience; (4) PRIDE helped enhance skills related to grant writing, networking, publishing, identifying mentors, presentation skills and developing research agendas, and long-term career plans; (5) PRIDE mentoring was unique—mentors shared experiences and engaged with mentees 1:1 and in groups	Future Directions: (1) Establishment of a program management tool (e.g., blackboard); (2) establishment of a step-by-step protocol for identifying external mentors; (3) incorporating strategies for negotiating a mentoring relationship; (4) long-term support beyond the PRIDE academic year
[66] Mancuso et al. (2019)	Design: Qualitative Purpose: Highlight faculty perceptions about mentoring Methods: Semi-structured interviews, grounded theory, constant comparative analytic strategy	Participants: 22 experienced research mentors from a variety of disciplines within academic medicine	Barrier: (1) Lack of time	Facilitators: (1) Match personality of mentor and mentee; (2) institutional acknowledgment of mentoring efforts; (3) continuing education relative to mentoring skills; (4) examine short- and long-term goals of mentee; (5) formalize a plan with template and regular check ins; (6) evaluate mentor and mentee	Limitations: (1) Featured 1 institution; (2) participants mentored trainees at junior ranks, so answers are specific to that rank; (3) focused only on research mentoring in academic medicine
[67] Manson (2016)	Design: Essay, editorial Purpose: Discuss how individual and institutional mentoring contributes to success of early stage investigators		Barriers: (1) Putting the majority of responsibility for success on the mentee’s individual skills (vs. also considering environment, resources, climate, and connections)	Facilitators: (1) Share mentoring plan with mentee’s supervisors; (2) work smarter—integrate work across teaching, research and service; (3) mentees and mentors developed sociograms of the structure and patterns of key group interactions, reflecting work relations, channels of influence and lines of communication; (4) evaluate	
[68] Martina et al. (2014)	Design: Descriptive Purpose: Describe experience in a Clinical Translational Science Award (CTSA) institution on development, implementation and evaluation of hybrid online mentoring curriculum for CTSA trainees Methods: Mentee completes Academic Career Development Plan (ADCP); mentor completed an online questionnaire within 6 weeks of completing program	Participants: 20 women and 73 men in academic medicine who completed the mentor course and served as mentors Results: Online format is useful; three strengths of this format: convenience, engagement, and financial sustainability; mentors valued the course regardless of experience		Facilitators: (1) Mentors who are accessible, engaged, supportive, and affirming; (2) limit number of mentees; (3) teaching and training of skills; (4) clarity of performance via an academic career development plan (ACDP); (5) sponsorship, share power and protect; (6) demystify academia; (7) challenge and encourage risk taking; (8) provide feedback; (9) self-disclosure; (10) affirm and nurture the dream	
[69] Masterson et al. (2019)	Design: Descriptive, quantitative, program evaluation Purpose: Evaluate Columbia University Mentor Peer Aging Research (CoMPAdRE) program for ESI using the Reach Effectiveness Adoption Implementation and Maintenance Framework (RE-AIM) Methods: Program developed using RE-AIM framework; effectiveness based on career successes obtained via survey	Participants: 15 post-doc and early career participants from 5 states across 6 medical specialties Results: 93% were federally funded as NIH PIs; 91% agreed or strongly agreed that the program was instrumental in helping them develop careers	Barriers: (1) Traditional dyadic model can be challenging due to scarcity of expertise in certain areas; (2) maintaining relationship over time is difficult—requires considerable investment by both partners; (3) difficulty balancing competing clinical, administrative and research demands	Facilitators: (1) Use a variety of mentoring models; (2) alignment around their primary research topic (i.e., aging); (3) small group size and frequent interaction with speakers—who talked freely about their career path; (4) teach leadership and executive skills not typically taught in doctoral work	Limitations: (1) Faculty across the nation participated, which made it more expensive; (2) lack of diversity in first year of cohort—addressed in 2nd year; (3) 13% attrition Future Directions: More than one mentor to assist a cadre of mentees
[70] Milburn et al. (2019)	Design: Descriptive, mixed methods Purpose: Describe a NIDA/NIH mentoring training program within the UCLA HIV/AIDS, Substance Abuse and Trauma Training program (HA-STTP) Methods: Short- and long-term assessments	Participants: 20 scholars with background in medicine, psychology, public health Evaluation: Regular surveys + 3 outside evaluators (annual review); each scholar assessed by mentors Results: > 70% submitted grant proposals and 1st authored papers		Facilitators: (1) Protected time; (2) first authored publications; (3) grants and serving on NIH study sections; (4) density of collaborative networks; (5) grounding curricula in cultural humility; (6) mentoring emerging colleagues; (7) significant planned time with mentors; (8) baseline assessment to plan sessions; (9) regular meetings	
[71] Ofili et al. (2013)	Design: Descriptive Purpose: Describe the mechanism CTSA created to foster formal collaborations between research intensive universities and MSIs Methods: Morehouse School of Medicine and Emory University proposed a CTSA/RCMI collaborative national mentoring model. Social network tools used to connect underrepresented students and ESI with experienced mentors	Participants: Emory, Morehouse Georgetown, Howard, UCLA, Charles Drew U, Vanderbilt, Meharry Medical College Cornell, Hunter College Results: Partnerships facilitate research and career development; multiple collaborative pilot studies, and participation in designing and implementing research programs is helpful		Facilitators: (1) Address health disparities together; (2) develop scientific methods through conferences; (3) provide funding when access to minority population is feasible or when rare disease is investigated; (4) form health disparities working groups; (5) promote more diverse participation in clinical research; (6) use interactive videoconferencing technologies for activities, and promote and maintain long-distance collaboration; (7) expand community partnerships	
[72] Pfund et al. (2016)	Design: Literature review Purpose: Propose core attributes of effective mentoring relationships, supported by literature, and suggested by theoretical models of academic persistence and propose ways to measure these variables Methods: Provide theoretical basis and ways to assess core attributes of effective mentoring in 5 categories: research, interpersonal, psychosocial and career, cultural responsiveness and diversity, and sponsorship	Participants: Emerging researchers from diverse populations		Facilitators: (1) Research: disciplinary and technical skills, ethical research, self-efficacy; (2) Interpersonal: active listening, align expectations, build trust; (3) Psychosocial and Career: motivation, coping, science identity, belonging; (4) Cultural Responsiveness and Diversity: equity and inclusion, cultural responsibility, reduce impact of bias and stereotypes; (5) Sponsorship: foster independence, promote prof. develop., grow networks, advocate	Future Directions: (1) Examine how mentoring complexities (e.g., race, ethnicity, gender) impact effectiveness; (2) develop assessments of effectiveness of research mentoring at various career stages; (3) conduct meta-analyses (based on theory)
[73] Redmond (2020)	Design: Presentation at NIH Regional Seminar (Oct 2020) Purpose: Define networking, identify networking benefits, potential members, opportunities and strategies			Facilitators: (1) Build relationships for emotional, instrumental, informational and appraisal support; (2) develop networks: navigator (org. dynamics), sponsor (networking), coach (prof. behavior), and confidant (listens); (3) find people who help get the job done, advance career goals, and provide personal support; (4) set and revisit goals, negotiate meeting frequency, establish desired modes of communication and fdbk. prefs.	
[74] Shea et al. (2011)	Design: Descriptive, mixed methods Purpose: Explore academic medical perspectives on how career development awardees are selected and mentored Methods: Survey administered at 2010 APM Winter meeting, followed by focus groups	Participants: Chairs of U.S. Internal Medicine Departments (*n* = 66) and Directors of CTSA centers (*n* = 23) (NOTE: These leaders are responsible for 25,000 junior faculty in academic medicine)		Facilitators: (1) Grant and manuscript writing and mgmt. workshops; (2) identify mentor before applying for funding; (3) seek bridge funding to move to independent funding; (4) important mentor roles: reviewing and editing, career negotiation and development, accountability and clear and timely communication; (5) number of pubs as senior author in high-impact journals; (6) passion	Limitations: Small sample; possible selection bias Future Directions: Better measures of mentoring effectiveness (test training and curricula); better rewards for mentoring
[75] Shiramizu et al. (2016)	Design: Descriptive, mixed methods Purpose: Success of Pilot Project Program (PPP) at U Hawaii RCTR for advancing careers of emerging investigators and commun. Collaborators Methods: Interviews with PPP investigators; analyzed infrastructure, awards, collaborations, advocacy and scientific impact, contributions made	Participants: 17/18 who received PPP participated in study Results: 17 PPP PIs had 47 grants (34 completed after receiving pilot funds)		Facilitators: (1) Assistance with grant administration; (2) prof. development opps.; (3) collaborations and partnerships; (4) access to biomedical informatics; (5) access to clinical research resources and facilities; (6) community-based research design and biostats; (7) regulatory knowledge and evaluation	
[76] Snyder-Mackler (2015)	Design: Descriptive, presentation, essay Purpose: Describe the barriers to entry into formal research training for DPT students and practicing PTs Methods: Award recipient presentation turned into manuscript		Barriers: (1) Educational debt; (2) desire to practice clinically; (3) lack of knowledge about conducting research studies; (4) mean percentage of time core PT faculty spend on scholarship is 20%; (5) “The only way to be sure you won’t get funded is to not submit!”; (6) heavy teaching requirements at non R1 institutions and within clinical programs	Facilitators: (1) Training grant to defray cost of education; (2) NIH Funded Comprehensive Opportunities in Rehabilitation Research Training (CORRT)—collective effort of 9 universities; (3) strategic alliances to survive competitive culture; (4) parental accommodation policy—so women/parents will not be dissuaded from academic life; (5) adjust teaching loads to include supervision of student research and independent scholarship; (6) strong team mentoring; (7) leadership that values research/mentoring, listens to ideas, and sets high expectations	
[77] Sorkness et al. (2017)	Design: Descriptive Purpose: Describe structure and activities of the National Research Mentoring Network (NRMN) Methods: NRMN activities (a) based on theory, (b) address bias, (c) focus on the preparation of mentors and mentees, (d) build community, and (e) include different modes of training Diversity Program Consortium (DPC) = BUILD = building infrastructure leading to diversity; CEC = coordination and evaluation center; NRMN = National Research Mentoring Network		Barriers: (1) UMF trainees receive less mentoring than their non-minority peers; (2) lack of understanding about institutional requirements and lack of institutional support; (3) failure to consider social, cultural and environmental factors that impact productivity—especially at white institutions (e.g., marginalization, overt/covert racism, involvement in non-career-enhancing activities)	Facilitators: (1) Align expectations between mentor and mentee; (2) emphasize communication; (3) address equity and inclusion; (4) theory-based (social cognitive theory) with attention to formation of science and cultural identity across faculty developmental stages; (5) authentically address bias, stereotype threat, and cultural ignorance; (6) focus on preparation of both mentors and mentees; (7) community building focus; (8) multimodal training formats	Future Directions: Need additional research to determine which types of mentoring relationships (dyads, peers) and which modes of mentoring (formal, informal) have the most impact on success, conditioned by context and career stage
[78] Stamatakis et al. (2013)	Design: Descriptive, quantitative Purpose: Outline perceived usefulness, importance of and barriers to developing as an ESI in dissemination and implementation research Methods: survey questions sent via email	Participants: 11 MD or PhD researchers with a background in mental health research		Facilitators: Mentors who help with: (1) developing links to practice settings (clinics and health depts) and collaborations with community partners; (2) balancing essential activities with those less valued by the academy; (3) training and technical assistance (e.g., grant writing, responding to reviewer feedback); (4) identifying productive research topics and providing data; (5) recommending supports (protected time, research assistants, committee burden, supervision of doctoral students, travel/training budget); (7) setting goals and being accountable	Limitations: Research is focused on U.S.-based faculty located in academic medical centers with traditional promotion policies Future Directions: Need to study impact of different styles of mentoring
[79] Stoff (2019)	Design: Descriptive, editorial Purpose: Describe mentoring programs that enhance diversity in the HIV research workforce, including the research education grant mechanism (R25) to promote new investigator development in HIV-related topics		Barriers: (1) Leaky pipeline—not knowing factors that influence ESI from diverse backgrounds to enter, exit and sustain a behavioral and biomedical career	Facilitators: (1) Community engagement and transdisciplinary team science; (2) technical expertise, career advice, and professional skills development (e.g, presentations, manuscripts, grants; leadership skills); (3) pilot projects with hands-on exposure to research; (4) leverage a variety of grants and mentors; (5) establish mentee proficiency in interdisciplinary competencies; (6) develop interinstitutional consortia partnerships	Limitations: Correlation data are limited in determining impact of R25 on publication/grant Future Directions: Analyze social networks for collaboration patterns; use theory to guide approaches; broaden pipeline of ESI backgrounds; propose asset models and leadership opportunities
[80] Sutton et al. (2013)	Design: Descriptive Purpose: Describe history and structure of Minority HIV/AIDS Research Initiative (MARI) and review data/accomplishments for MARI which supports underrepresented minority scientists performing HIV research in affected communities Methods: Review MARI impact from 2003 to 2013	Participants: 27 scientist leaders who have HIV prevention interventions Results: Accomplishments: developed research programs in communities of color, obtaining more funding for research and programmatic work		Facilitators: (1) Release time for mentees and mentors; (2) forming a collaborative network of mentors in HIV prevention research; (3) sustained institutional and financial support of historically underrepresented scientists	
[81] Sweeney et al. (2017)	Design: QT, descriptive Purpose: Describe transition from mentored to independent research funding for clinical and translational scholars supported by institutional KL2 Mentored Career Develop. Programs Methods: Online survey examining characteristics of KL2 scholars in their from 2006 to 2013. Primary outcome variable was whether scholar had received independent funding as PI.	Participants: 48 respondents from institutions providing information about 914 KL2 scholars Results: 68% MDs, 19% non-clinician PhD, 53% female, 12% URM; amount of NIH funding an institution received was not predictive of an individual’s success transitioning to indep. research funding	Barriers: (1) Clinicians without a PhD are less likely to have independent funding after program	Facilitators: (1) Mentoring/support to scholars through CTSA core facilitates success of female and URM awardees compared with individual CDA mechanisms; (2) having a PhD at the time of KL2 appointment was positively associated with attaining independent funding	Future Directions: The NIH should consider a longer mentored program that combines KL2 training and K08/K23 training to fully prepare clinician-scholars for independent funding
[82] Teruya et al. (2013)	Design: Literature review Purpose: Review faculty development programs and competencies (type, components, outcomes, limitations) Methods: Review of 19 mentoring intervention studies published in English between 2004 and 2013	Participants: Researchers in biomedical sciences; 17 of 19 studies conducted in US and Puerto Rico		Facilitators: (1) Mentoring and guided or participatory learning = most successful; (2) variety of delivery methods for workshops was more enjoyable	Limitations: Info. delivered via lectures; no control group; hard to determine impact of prior research experience Future Research: Need prospective studies w/participants who are randomized and receive intervention or control; assessment w/standard, objective criteria
[83] Thorpe et al. (2020)	Design: Descriptive, quantitative Purpose: Assess program impact on grant writing self- efficacy Methods: Longitudinal, experimental study of 12 month intervention w/longitudinal follow up using grant self-efficacy scale focused on conceptualizing, designing and funding a grant	Participants: Trainees were post-docs, or ESI, mostly Black (62%), female (62%); Asst Profs (52%); 24% had no post-doc training		Facilitators: (1) After training, grant self-efficacy improved on all 3 domains	Limitations: Small sample size Future Directions: Need to study if increasing grant SE will translate to increased grant proficiency and productivity later in career
[84] Varkey et al. (2012)	Design: Descriptive, quantitative Purpose: Study the impact of facilitated peer mentoring on scholarly output (manuscripts submitted) and the impact of peer mentoring on self-efficacy of writing skills Methods: Longitudinal prog. eval. of a 12 month prog. with peer mentor groups and experienced faculty facilitating. Met every 2–4 weeks, to review and edit drafts, w/individual and project mentoring. Initial self-assessment repeated at end	Participants: 21 women faculty in Department of Medicine at Mayo Clinic holding rank of Instructor or Assistant professor Results: at end of 12 month project, manuscripts/grants increased + significant changes in satisfaction with academic accomplishments and confidence and motivates to accomplish goals	Barriers: (1) Program too short; (2) difficult to find time to meet	Facilitators: (1) Protected time for research; (2) having a good mentor; (3) introduction to broad scope of resources available; (4) mentors and mentees reported benefits; (5) identify plans to achieve career goals	Limitations: Long-term outcomes and sustainability not evaluated; project; did not compare faculty who participated to those who did not; program may have self-selected highly motivated individuals Future Directions: Extend duration of intervention and assess long-term outcomes
[85] Velasquez et al. (2019)	Design: Descriptive, quantitative Purpose: Survey awardees of the Minority HIV Investigator Mentoring Program (MHIMP) of the AIDS Clinical Trials Group Methods: Longitudinal evaluation of a 1 year program to help minority junior investigators jumpstart their careers as HIV investigators. Mentees choose a mentor, outline a 12 month career development plan and prepare a research proposal. Survey monkey platform delivered 35Q survey	Participants: 22/31 participants from 1996 to 2017 completed the survey Results: All but 1 performing medical or health sciences research, with 55% involved in HIV/AIDS or viral research; 91% had research funding (73% as PI); 95% mentor others in HIV-related research		Facilitators: (1) Provide 25% of mentee and 2.5% of mentor salary; (2) involvement with existing network of established investigators; (3) gain experience on various national scientific committees, which provided exposure to protocol development, study team structure and networking	Future Directions: (1) Cost–benefit analysis to determine whether increasing quantity and duration of support results in larger impact; (2) include other underrepresented populations (sexual and gender minorities, people with disabilities); (3) examine UMF at different career stages
[86] Vermund et al. (2018)	Design: Descriptive, evaluation Purpose: Evaluate HIV Prevention Trials Network (HPTN) that mentors early career investigators from underrepresented minority groups Methods: Describe program and conduct exit evaluations with alumni from cohorts 1, 2, and 3 (benefits of program 2–4 years after completing it)	Participants: 26 Research and Mentorship Program (RAMP) medical students who conducted either summer (2–4 months) or 9–12 month projects within the HIV Vaccine Trials Network (HVTN).	Barriers: (1) Programs shorter than 12 months—most felt a longer program would be beneficial; (2) isolation in home institutions; (3) failure to connect ESI and UMF with senior mentors	Facilitators: (1) % of mentee’s annual salary, funding for research supplies, travel paid; (2) mentors help complete projects, develop knowledge, skills and connections; (3) mentor and mentee matched; (4) regular interactions; (5) facilitate mentees contacting NIH institute leadership and collaborators	
[87] Vishwanatha and Jones (2018)	Design: Descriptive, mixed methods Purpose: Report program design, curricula, outcomes in preparing UMF and community partners for careers in health disparity research Methods: 12 month program delivered remotely and F2F. Fellows attend workshops for skill development, and speak with mentor every two weeks	Participants: 71 total national fellows as of 2016 Results: 65% female, 48% Black, 20% Asian, 10% Hispanic, 39% from MSI. Increase in publications; over $6 million in funding (ave. per fellow = $90k; 93% rated the program as excellent or very good		Facilitators: (1) Participated in online activities; (2) received support from dean, chair, or supervisor; (3) “research readiness” of fellows assessed based on self-beliefs; (4) learned principles of research and health disparities; (5) focused on community-based participatory research (CBPR); (6) matched mentor and mentee	
[88] Zambrana et al. (2015)	Design: Qualitative Purpose: To highlight the importance of mentoring, reflect on when mentoring is absent, and examine ideal attributes of mentoring relationships, and challenges to effective mentoring Methods: Network sampling to identify participants; interviews and focus groups conducted drawing on a dual conceptual framework: intersectionality and social capital; Atlas.ti used for coding, analysis and interpretation	Participants: 58 UMF at 22 RI institutions Results: 38% from social sciences and 32% in STEM, health or medicine; most frequent activities: opps. for collaboration, coauthoring articles, invitations to present at conferences and an annual career review; 25% said poor mentoring hindered growth	Barriers: (1) Undervaluing faculty research areas and CBPR; (2) overcoming “imposter syndrome”; (3) patchwork of mentors (rather than systematic and intentional mentors); (4) supervisory rather than advisory interactions; (5) mentors with little familiarity or interest in their areas of research; (6) failure of a mentor to understand CBPR	Facilitators: (1) Mentors who understand struggles of UMF at predominantly white institutions; (2) support of research focus on marginalized populations; (3) mentor who will critique and edit work; (4) multiple mentors with different skills to serve different needs—with some located at different institutions to provide a “safe space” for discussions about the home institution	Limitations: (1) Small sample and possible selection and social desirability bias—study limited to those who volunteered; (2) cross-sectional design; (3) Native American scholars not included

**Table 2:** Summary of individual barriers to research success for new or early-stage investigators and/or underrepresented minority faculty.

[Ref] Study Author(s) (Year)	Study Characteristics ^a^	Bias and Discrimination (*n* = 5)	Isolation (*n* = 5)	Lack of Time (*n* = 5)	Find Work–Life Balance (*n* = 4)
[44] Beech et al. (2020)	D, QL, ESI, NI, UMF, MSI	x			
[45] Berget et al. (2010)	D, MM, ESI, NI, UMF, PA	x	x		
[52] Cross et al. (2019)	LR, NI, ESI			x	
[53] Cruz et al. (2020)	QL, NI, ESI, UMF			x	
[55] Duncan et al. (2016)	QL, NI, ESI, UMF				
[57] Espino and Zambrana (2019)	MM, NI, ESI, UMF, RO			x	
[58] Felder et al. (2019)	QT, NI, ESI, UMF				x
[60] Flores et al. (2016)	D, NI, ESI, UMF	x	x		
[65] Jean-Louis et al. (2016)	MM, NI, ESI, UMF	x	x		
[66] Mancusco et al. (2019)	QL, NI, ESI			x	
[69] Masterson et al. (2019)	QT, NI, ESI			x	x
[76] Snyder-Mackler (2015)	P, NI, ESI				x
[77] Sorkness et al. (2017)	D, NI, ESI, UMF	x			
[81] Sweeney et al. (2017)	MM, D, NI, ESI				x
[86] Vermund et al. (2018)	QT, NI, ESI, UMF		x		
[88] Zambrana et al. (2015)	D, QL, NI, ESI, UMF, RO		x		

^a^**Type of study:** D = descriptive, E = essay or editorial, LR = literature review, MM = mixed-methods research (qualitative and quantitative), P = presentation, QL = qualitative research, and QT = quantitative research. **Study participants:** ESI = focus of study primarily on early-stage investigator, NI = focus of study primarily on new investigator, and UMF = focus of study primarily on underrepresented minority faculty. **Institution type:** MSI = minority serving institution (includes historically Black colleges and universities), RO = research oriented, and PA = partnership between MSI and RO college or university.

**Table 3:** Summary of institutional barriers to research success for new or early-stage investigators and/or underrepresented faculty.

[Ref.] Study Author(s) (Year)	Study Characteristics ^a^	Lack of Mentors (*n* = 12)	Lack of Access to Resources (*n* = 9)	Heavy Teaching and Service Load (*n* = 7)	Bias (*n* = 6)
[44] Beech et al. (2020)	D, QL, ESI, NI, UMF, MSI	x	x	x	x
[45] Berget et al. (2010)	D, MM, ESI, NI, UMF, PA	x	x	x	x
[46] Brewer et al. (2016)	QL, NI, ESI, UMF	x	x	x	
[48] Byington et al. (2016)	D, NI, ESI, RO		x		
[52] Cross et al. (2019)	LR, NI, ESI	x			
[55] Duncan et al. (2016)	QL, NI, ESI, UMF	x	x	x	
[57] Espino and Zambrana (2019)	MM, NI, ESI, UMF, RO				x
[60] Flores et al. (2016)	D, NI, ESI, UMF				
[63] Harawa et al. (2017)	MM, NI, ESI, UMF	x			
[64] Hemming et al. (2019)	D, NI, ESI, UMF	x	x	x	
[65] Jean-Louis et al. (2016)	MM, NI, ESI, UMF		x		x
[67] Manson et al. (2016)	E, ESI	x	x	x	
[69] Masterson et al. (2019)	QT, NI, ESI	x			
[76] Snyder-Mackler (2015)	P, NI, ESI			x	
[77] Sorkness et al. (2017)	D, NI, ESI, UMF	x	x		x
[79] Stoff (2019)	E, NI, ESI, UMF				
[86] Vermund et al. (2018)	QT, NI, ESI, UMF	x			
[88] Zambrana et al. (2015)	D, QL, NI, ESI, UMF, RO	x			x

^a^**Type of study:** D = descriptive, E = essay or editorial, LR = literature review, MM = mixed-methods research (qualitative and quantitative), P = presentation, QL = qualitative research, and QT = quantitative research. **Study participants:** ESI = focus of study primarily on early-stage investigator, NI = focus of study primarily on new investigator, and UMF = focus of study primarily on underrepresented minority faculty. **Institution type:** MSI = minority serving institution (includes historically Black colleges and universities), RO = research oriented, and PA = partnership between MSI and RO college or university.

**Table 4:** Summary of technical skills recommended for new faculty, early career faculty, and underrepresented minority faculty.

[Ref.] Study Author(s) (Year)	Study Characteristics ^a^	Writing (*n* = 17)	Analytical Skills (*n* = 4)	Presentation Skills (*n* = 4)
[45] Berget et al. (2010)	D, MM, ESI, NI, UMF, PA	x		
[43] Blanchard et al. (2019)	MM, NI, ESI, UMF	x		
[47] Buist et al. (2017)	D, NI, ESI	x		x
[48] Byington et al. (2016)	D, NI, ESI, RO	x	x	
[54] Doyle et al. (2019)	LR, NI, ESI		x	
[59] Feldman et al. (2010)	D, QT, NI, ESI, RO	x		x
[62] Flores et al. (2019)	D, NI, ESI, UMF	x		
[65] Jean-Louis et al. (2016)	MM, NI, ESI, UMF	x		x
[70] Milburn et al. (2019)	D, MM, NI, ESI	x		
[72] Pfund et al. (2016)	LR, NI, ESI, UMF	x	x	
[73] Redmond (2020)	P, NI, ESI	x		
[74] Shea et al. (2011)	D, MM, NI, ESI	x		
[75] Shiramizu et al. (2016)	D, MM, NI, ESI, UMF	x		
[78] Stamatakis et al. (2013)	D, QT, NI, ESI	x	x	
[79] Stoff (2019)	E, NI, ESI, UMF	x		x
[83] Thorpe et al. (2020)	QT, UMF, RO	x		
[86] Vermund et al. (2018)	QT, NI, ESI, UMF	x		
[87] Vishwanatha and Jones (2018)	D, MM, NI, ESI, UMF, MSI	x		

^a^**Type of study:** D = descriptive, E = essay or editorial, LR = literature review, MM = mixed-methods research (qualitative and quantitative), P = presentation, QL = qualitative research, and QT = quantitative research. **Study participants:** ESI = focus of study primarily on early-stage investigator, NI = focus of study primarily on new investigator, and UMF = focus of study primarily on underrepresented minority faculty. **Institution type:** MSI = minority serving institution (includes historically Black colleges and universities), RO = research oriented, and PA = partnership between MSI and RO college or university.

**Table 5:** Summary of interpersonal skills recommended for new faculty, early career faculty, and underrepresented minority faculty.

[Ref.] Study Author(s) (Year)	Study Characteristics ^a^	Finding Productive Collaborators (*n* = 33)	Networking (*n* = 28)	Advocacy for Diversity and Cultural Humility (*n* = 12)	Managing Data, Projects and Teams (*n* = 7)	Organizational Dynamics and Navigating Political Traps (*n* = 6)
[44] Beech et al. (2020)	D, QL, ESI, NI, UMF, MSI	x				
[45] Berget et al. (2010)	D, MM, ESI, NI, UMF, PA		x			
[43] Blanchard et al. (2019)	MM, NI, ESI, UMF	x				
[46] Brewer et al. (2016)	QL, UMF		x			x
[47] Buist et al. (2017)	D, NI, ESI				x	
[48] Byington et al. (2016)	D, NI, ESI, RO	x	x		x	
[51] Comeau et al. (2017)	MM, NI, ESI, UMF	x	x			
[53] Cruz et al. (2020)	QL, NI, SI, UMF	x				
[55] Duncan et al. (2016)	QL, NI, ESI, UMF	x	x	x		
[57] Espino and Zambrana (2019)	MM, NI, ESI, UMF, RO	x	x	x		
[59] Feldman et al. (2010)	D, QT, NI, ESI, RO	x	x			
[60] Flores et al. (2016)	D, NI, ESI, UMF	x	x	x		
[62] Flores et al. (2019)	D, NI, ESI, UMF	x	x	x	x	
[63] Harawa et al. (2017)	MM, NI, ESI, UMF	x	x			x
[64] Hemming et al. (2019)	D, NI, ESI, UMF	x	x			
[65] Jean-Louis et al. (2016)	MM, NI, ESI, UMF	x	x	x		x
[66] Mancusco et al. (2019)	QL, NI, ESI	x				
[68] Martina et al. (2014)	D, NI, ESI	x	x			
[69] Masterson et al. (2019)	QT, NI, ESI	x	x			
[70] Milburn et al. (2019)	D, MM, NI, ESI	x	x	x		
[71] Ofili et al. (2013)	D, NI, ESI, UMF, PA	x	x	x		
[72] Pfund et al. (2016)	LR, NI, ESI, UMF		x	x		
[73] Redmond (2020)	P, NI, ESI	x	x			x
[74] Shea et al. (2011)	D, MM, NI, ESI	x				
[75] Shiramizu et al. (2016)	D, MM, NI, ESI, UMF	x	x		x	
[76] Snyder-Mackler (2015)	P, NI, ESI	x	x			x
[77] Sorkness et al. (2017)	D, NI, ESI, UMF	x		x		
[78] Stamatakis et al. (2013)	D, QT, NI, ESI	x	x			
[79] Stoff (2019)	E, NI, ESI, UMF	x	x		x	
[80] Sutton et al. (2013)	D, NI, ESI, UMF	x	x	x		
[81] Sweeney et al. (2017)	MM, D, NI, ESI	x				
[82] Teruya et al. (2013)	LR, NI, ESI	x				
[84] Varkey et al. (2012)	QT, NI, ESI	x	x			
[85] Velasquez et al. (2019)	QT, NI, ESI, UMF	x	x			
[86] Vermund et al. (2018)	QT, NI, ESI, UMF	x	x			
[87] Vishwanatha and Jones (2018)	MM, D, NI, ESI, UMF, MSI	x	x	x	x	
[88] Zambrana et al. (2015)	QL, D, NI, ESI, UMF, RO	x	x	x	x	x

^a^**Type of study:** D = descriptive, E = essay or editorial, LR = literature review, MM = mixed-methods research (qualitative and quantitative), P = presentation, QL = qualitative research, and QT = quantitative research. **Study participants:** ESI = focus of study primarily on early-stage investigator, NI = focus of study primarily on new investigator, and UMF = focus of study primarily on underrepresented minority faculty. **Institution type:** MSI = minority serving institution (includes historically Black colleges and universities), RO = research-oriented, and PA = partnership between MSI and RO college or university.

**Table 6:** Summary of personal skills recommended for new faculty, early career faculty, and underrepresented minority faculty.

[Ref.] Study Author(s) (Year)	Study Characteristics ^a^	Accountability (*n* = 17)	Career Planning (*n* = 14)	Leadership (*n* = 4)
[47] Buist et al. (2017)	D, NI, ESI			x
[48] Byington et al. (2016)	D, NI, ESI, RO	x		
[54] Doyle et al. (2019)	LR, NI, ESI	x		
[56] Efstanthiou et al. (2018)	MM, NI, ESI	x		
[57] Espino and Zambrana (2019)	MM, NI, ESI, UMF, RO	x		
[59] Feldman et al. (2010)	D, QT, NI, ESI, RO		x	
[61] Flores et al. (2020)	D, NI, ESI, UMF	x	x	
[62] Flores et al. (2019)	D, NI, ESI, UMF	x	x	
[63] Harawa et al. (2017)	MM, NI, ESI, UMF	x		
[65] Jean-Louis et al. (2016)	MM, NI, ESI, UMF		x	
[66] Mancusco et al. (2019)	QL, NI, ESI	x	x	
[67] Manson (2016)	E, ESI	x		
[68] Martina et al. (2014)	D, NI, ESI	x	x	
[69] Masterson et al. (2019)	QT, NI, ESI		x	x
[72] Pfund et al. (2016)	LR, NI, ESI, UMF		x	
[73] Redmond (2020)	P, NI, ESI	x	x	
[74] Shea et al. (2011)	D, MM, NI, ESI	x		
[76] Snyder-Mackler (2015)	P, NI, ESI	x	x	
[78] Stamatakis et al. (2013)	D, QT, NI, ESI	x		
[79] Stoff (2019)	E, NI, ESI, UMF		x	x
[84] Varkey et al. (2012)	QT, NI, ESI	x	x	
[86] Vermund et al. (2018)	QT, NI, ESI, UMF	x	x	x
[87] Vishwanatha and Jones (2018)	MM, D, NI, ESI, UMF, MSI	x	x	

^a^**Type of study:** D = descriptive, E = essay or editorial, LR = literature review, MM = mixed-methods research (qualitative and quantitative), P = presentation, QL = qualitative research, and QT = quantitative research. **Study participants:** ESI = focus of study primarily on early-stage investigator, NI = focus of study primarily on new investigator, and UMF = focus of study primarily on underrepresented minority faculty. **Institution type:** MSI = minority serving institution (includes historically Black colleges and universities), RO = research-oriented, and PA = partnership between MSI and RO college or university.

**Table 7:** Summary of institutional facilitators recommended for new faculty, early career faculty, and underrepresented minority faculty.

[Ref.]	Study Characteristics ^a^	Access to Expertise and Mentoring (*n* = 45)	Prof. Development Opportunities (*n* = 38)	Science Culture (*n* = 17)	Workload Assigned to Research (*n* = 16)	Culturally Responsive Institution and Mentoring Strategies (*n* = 16)	Funding, Equipment and Facilities (*n* = 14)	Pre-Assess Research Skills (*n* = 6)
[44]	D, QL, ESI, NI, UMF, MSI	x	x		x	x	x	
[45]	D, MM, ESI, NI, UMF, PA	x	x					
[43]	MM, NI, ESI, UMF	x	x					
[46]	QL, UMF	x	x		x	x		
[47]	D, NI, ESI	x	x	x	x			x
[48]	D, NI, ESI, RO	x	x	x				
[49]	D, UMF, PA		x	x				
[50]	QT, NI, ESI	x	x	x	x			
[51]	MM, NI, ESI, UMF	x	x	x	x			
[52]	LR, NI, ESI	x				x		
[53]	QL, NI, ESI, UMF	x	x			x		
[54]	LR, NI, ESI	x		x				
[55]	QL, NI, ESI, UMF	x	x			x	x	
[56]	MM, NI, ESI	x	x					x
[57]	MM, NI, ESI, UMF, RO	x				x		
[58]	QT, NI, ESI, UMF	x				x		
[59]	D, QT, NI, ESI, RO	x	x					
[60]	D, NI, ESI, UMF	x	x		x	x		
[61]	D, NI, ESI, UMF	x	x				x	
[62]	D, NI, ESI, UMF	x	x			x		
[63]	MM, NI, ESI, UMF	x	x					
[64]	D, NI, ESI, UMF	x	x	x	x		x	
[65]	MM, NI, ESI, UMF	x	x			x		
[66]	QL, NI, ESI	x	x		x			
[67]	E, ESI	x	x			x		
[68]	D, NI, ESI	x	x					x
[69]	QT, NI, ESI	x	x					
[70]	D, MM, NI, ESI	x	x	x	x	x		x
[71]	D, NI, ESI, UMF	x	x	x		x		
[72]	LR, NI, ESI, UMF	x		x		x		
[73]	P, NI, ESI	x						
[74]	D, MM, NI, ESI	x	x	x			x	
[75]	D, MM, NI, ESI, UMF	x	x				x	
[76]	P, NI, ESI	x	x	x	x			
[77]	D, NI, ESI, UMF	x	x			x		
[78]	D, QT, NI, ESI	x	x		x		x	
[79]	E, NI, ESI, UMF	x	x	x			x	
[80]	D, NI, ESI, UMF	x	x	x	x		x	
[81]	MM, D, NI, ESI	x						
[82]	LR, NI, ESI	x	x	x			x	
[84]	QT, NI, ESI	x	x	x	x		x	x
[85]	QT, NI, ESI, UMF	x	x	x	x		x	
[86]	QT, NI, ESI, UMF	x	x		x		x	
[87]	MM, D, NI, ESI, UMF, MSI	x	x		x			x
[88]	QL, D, NI, ESI, UMF, RO	x	x			x	x	

^a^**Type of study:** D = descriptive, E = essay or editorial, LR = literature review, MM = mixed-methods research (qualitative and quantitative), P = presentation, QL = qualitative research, and QT = quantitative research. **Study Participants:** ESI = focus of study primarily on early-stage investigator, NI = focus of study primarily on new investigator, and UMF = focus of study primarily on underrepresented minority faculty. **Institution type:** MSI = minority serving institution (includes historically Black colleges and universities), RO = research-oriented, and PA = partnership between MSI and RO college or university.

## Data Availability

No new data were created in this study. Data sharing is not applicable to this article.

## References

[1] Cote J.E., Allahar A.L. (2015). Lowering Higher Education: The Rise of Corporate Universities and the Fall of Liberal Education.

[2] Grawe N.D. (2018). Demographics and the Demand for Higher Education.

[3] Blumenstyk G. (2015). American Higher Education in Crisis? What Everyone Needs to Know.

[4] Dennis M.J. (2020). Post-COVID-19 threats to higher education. Enroll. Manag. Rep..

[5] Bucklin B.A., Valley M., Welch C., Tran Z.V., Lowenstein S.R. (2014). Predictors of early faculty attrition at one academic medical center. BMC Med. Educ..

[6] American Association of University Professors (AAUP) Tenure. https://www.aaup.org/issues/tenure.

[7] Nikaj S., Roychowdhury D., Lund P.K., Matthews M., Pearson K. (2018). Examining trends in the diversity of the U.S. National Institutes of Health participating and funded workforce. FASEB J..

[8] Franco I., Bailey L.O., Bakos A.D., Springfield S.A. (2011). The continuing umbrella of research experiences (CURE): A model for training underserved scientists in cancer research. J. Cancer Educ..

[9] Kosoko-Lasaki O., Sonnino R.E., Voytko M.L. (2006). Mentoring for women and underrepresented minority faculty and students: Experience at two institutions of higher education. J. Natl. Med. Assoc..

[10] TIAA Institute Taking the Measure of Faculty Diversity. https://www.tiaainstitute.org/sites/default/files/presentations/2017-02/taking_the_measure_of_faculty_diversity.pdf.

[11] National Institutes of Health (NIH) Populations Underrepresented in the Extramural Scientific Workforce. https://diversity.nih.gov/about-us/population-underrepresented.

[12] Brown S.E., Takahashi K., Roberts K.D. (2010). Mentoring individuals with disabilities in postsecondary education: A review of the literature. J. Postsec. Educ. Disabil..

[13] Haeger H., Fresquez C. (2016). Mentoring for inclusion: The impact of mentoring on undergraduate researchers in the sciences. CBE Life Sci. Educ..

[14] Nardi D.A., Gyurko C.C. (2013). The global nursing faculty shortage: Status and solutions for change. J. Nurs. Sch..

[15] Carnevale A.P., Smith N., Gulish A. (2018). Nursing: A closer look at workforce opportunities, education, and wages. Am. J. Med. Res..

[16] Girod S.C., Fassiotto M., Menorca R., Etzkowitz H., Wren S.M. (2017). Reasons for faculty departures from an academic medical center: A survey and comparison across faculty lines. BMC Med. Educ..

[17] Primack B.A., Dilmore T.C., Switzer G.E., Bryce C.L., Seltzer D.L., Li J., Landsittel D.P., Kapoor W.N., Rubio D.M. (2010). Burnout among early career clinical investigators. Clin. Transl. Sci..

[18] American Psychological Association Introduction to Mentoring: A Guide for Mentors and Mentees. https://www.apa.org/education/grad/mentoring.

[19] Mullen C.A., Hutinger J.L. (2008). At the tipping point? Role of formal faculty mentoring in changing university research cultures. J. In-Service Educ..

[20] McRae M.P., Zimmerman K.M. (2019). Identifying components of success within health sciences-focused mentoring programs through a review of the literature. Am. J. Pharm. Educ..

[21] Nowell L., Norris J.M., Mrklas K., White D.E. (2016). Mixed methods systematic review exploring mentorship outcomes in nursing academia. J. Adv. Nurs..

[22] Allen T.D., Eby L.T., Poteet M.L., Lentz E., Lima L. (2004). Career benefits associated with mentoring for proteges: A meta-analysis. J. Appl. Psychol..

[23] Eby L.T., Allen T.D., Evans S.C., Ng T., DuBois D. (2008). Does mentoring matter? A multidisciplinary meta-analysis comparing mentored and non-mentored individuals. J. Vocat. Behav..

[24] Eby L.T., Allen T.D., Hoffman B.J., Baranik L.E., Sauer J.B., Baldwin S., Morrison M.A., Kinkade K.M., Maher C.P., Curtis S. (2013). An interdisciplinary meta-analysis of the potential antecedents, correlates and consequences of protégé perceptions of mentoring. Psychol. Bull..

[25] Ransdell L.B., Nguyen N., Hums M., Clark M., Williams S.B. (2017). Voices from the field: Perspectives of U.S. Kinesiology chairs on opportunities, challenges, and the role of mentoring in the chair position. Quest.

[26] Ransdell L.B. (2014). Women as leaders in kinesiology and beyond: Strategies for breaking through the glass obstacles. Quest.

[27] Knowles S. (2020). Initiative of a mentoring program: Mentoring invisible nurse faculty. Teach. Learn. Nurs..

[28] Phillips W.R. (2018). Pursuing personal passion: Learner-centered research mentoring. Fam. Med..

[29] Jokelainen M., Turunen H., Tossavainen K., Jamookeeah D., Coco K. (2011). A systematic review of mentoring nursing students in clinical placements. J. Clin. Nurs..

[30] Moran A.M., Coyle J., Pope R., Boxall D., Nancarrow S.A., Young J. (2014). Supervision, support and mentoring interventions for health practitioners in rural and remote contexts: An integrative review and thematic synthesis of the literature to identify mechanisms for successful outcomes. Hum. Resour. Health.

[31] Sng J.H., Pei Y., Toh Y.P., Peh T.Y., Neo S.H., Krishna L.K.R. (2017). Mentoring relationships between senior physicians and junior doctors and/or medical students: A thematic review. Med. Teach..

[32] Windsor J., Garrod T., Talley N.H., Tebbutt C., Churchill J., Farmer E., Baur L., Smith J.A. (2017). The clinical academic workforce in Australia and New Zealand: Report on the second binational summit to implement a sustainable training pathway. Intern. Med. J..

[33] Scala E., Price C., Day J. (2016). An integrative review of engaging clinical nurses in nursing research. J. Nurs. Sch..

[34] Budderberg-Fischer B., Herta K.D. (2006). Formal mentoring programmes for medical students and doctors—A review of the Medline literature. Med. Teach..

[35] Byrne M.W., Keefe M.R. (2002). Building research competence in nursing through mentoring. J. Nurs. Sch..

[36] White M.T., Satterfield C.A., Blackard J.T. (2017). Essential competencies in global health research for medical trainees: A narrative review. Med. Teach..

[37] Chua W.J., Shuen C.W., Lee F.Q.H., Koh E.Y.H., Tohn Y.P., Mason S., Krishna L.K.R. (2020). Structuring mentoring in medicine and surgery. A systematic scoping review of mentoring programs between 2000 and 2019. J. Contin. Educ. Health Prof..

[38] Lefebvre J.S., Bloom G.A., Loughead T.M. (2020). A citation network analysis of career mentoring across disciplines: A roadmap for mentoring research in sport. Psychol. Sport Exerc..

[39] Whittemore R., Knafl K. (2005). The integrative review: Updated methodology. J. Adv. Nurs..

[40] Hassouneh D., Lutz K.F., Beckett A.K., Junkins E.P., Horton L.L. The experiences of underrepresented minority faculty in schools of medicine. Med. Educ. Online.

[41] Coe C., Piggott C., Davis A., Hall M.N., Goodell K., Joo P., South-Paul J.E. (2020). Leadership pathways in academic family medicine: Focus on underrepresented minorities and women. Fam. Med..

[42] Xu Y.J. (2008). Gender disparity in STEM disciplines: A study of faculty attrition and turnover intentions. Res. High. Educ..

[43] Blanchard S.A., Rivers R., Martinez W., Agodoa L. (2019). Building the network of minority health research investigators: A novel program to enhance leadership and success of underrepresented minorities in biomedical research. Ethn. Dis..

[44] Beech B.M., Norris K.C., Thorpe R.J., Heitman E., Marino B. (2020). Conversation Cafés and Conceptual framework formation for research training and mentoring of underrepresented faculty at Historically Black Colleges and Universities: Obesity Health Disparities (OHD) PRIDE program. Ethn. Dis..

[45] Berget R.J., Reynolds C.F., Ricci E.M., Quinn S.C., Mawson A.R., Payton M., Thomas S.B., Berget R.J., Reynolds C.F., Ricci E.M. (2010). A plan to facilitate the early career development of minority scholars in the health sciences. Soc. Work Public Health.

[46] Brewer R.A., Dyer T., Watson C.C., Scott H. (2016). Navigating opportunities, learning and potential threats: Mentee perspectives on mentoring in HIV research. AIDS Behav..

[47] Buist D.S., Field T.S., Banegas M.P., Clancy H.A., Doria-Rose V.P., Epstein M.M., Greenlee R.T., McDonald S., Nichols H.B., Pawloski P.A. (2017). Training in the conduct of population-based multi-site and multi-disciplinary studies: The Cancer Research Network’s scholars program. J. Cancer Educ..

[48] Byington C.L., Keenan H., Phillips J.D., Childs R., Wachs E., Berzins M.A., Clark K., Torres M.K., Abramson J., Lee V. (2016). A matrix mentoring model that effectively supports clinical and translational scientists and increases inclusion in biomedical research: Lessons from the University of Utah. Acad. Med..

[49] Campbell A.G., Leibowitz M.J., Murray S.A., Burgess D., Denetclaw W.F., Carrero-Martinez F.A., Asai Schinske D.J. (2013). Partnered research experiences for junior faculty at minority-serving institutions enhance professional success. CBE Life Sci. Educ..

[50] Cohen J.G., Sherman A.E., Kiet T.K., Kapp D.S., Osann K., Chen L.M., O’Sullivan P.S., Chan J.K. (2012). Characteristics of success in mentoring and research productivity—A case-control study of academic centers. Gynecol. Oncol..

[51] Comeau D.C., Escoffery C., Freedman A., Ziegler T.R., Blumberg H.M. (2017). Improving clinical and translational research training: A qualitative evaluation of the Atlanta Clinical and Translational Science Institute (ACTSI) KL2-mentored research scholars program. J. Investig. Med..

[52] Cross M., Lee S., Bridgman H., Thapa D.K., Cleary M., Kornhaber R. (2019). Benefits, barriers and enablers of mentoring female health academics: An integrative review. PLoS ONE.

[53] Cruz T.H., Borrego M.E., Page-Reeves J. (2020). Increasing the number of underrepresented minority behavioral health researchers partnering with underresourced communities: Lessons learned from a pilot research project program. Health Promot. Pract..

[54] Doyle N.W., Lachter L.G., Jacobs K. (2019). Scoping review of mentoring research in the occupational therapy literature, 2002–2018. Aust. Occup. Ther. J..

[55] Duncan G.A., Lockett A., Villegas L.R., Almodovar S., Gomez J.L., Flores S.C., Wilkes D.S., Tigno X.T. (2016). National Heart, Lung, and Blood Institute workshop summary: Enhancing opportunities for training and retention of a diverse biomedical workforce. Ann. Am. Thorac. Soc..

[56] Efstathiou J.A., Drumm M.R., Paly J.P., Lawton D.M., O’Neill R.M., Niemierko A., Leffert L.R., Loeffler J.S., Shih H.A. (2018). Long-term impact of a faculty mentoring program in academic medicine. PLoS ONE.

[57] Espino M.M., Zambrana R.E. (2019). “How do you advance here? How do you survive?” An exploration of underrepresented minority (URM) faculty perceptions of mentoring modalities. Rev. High. Ed..

[58] Felder T.M., Braun K.L., Brandt H.M., Khan S., Tanjasiri S., Friedman D.B., Armstead C.A., Okuyemi K.S., Hebert J.R. (2015). Mentoring and training of a cancer-related health disparities researchers committed to community-based participatory research. Prog. Community Health Partnersh..

[59] Feldman M.D., Arean P.A., Marshall S.J., Lovett M., O’Sullivan P. (2010). Does mentoring matter: Results from a survey of faculty mentees at a large health sciences university. Med. Educ. Online.

[60] Flores G., Mendoza F.S., Fuentes-Afflick E., Mendoza J.A., Pachter L., Espinoza J., Fernandez C.R., Arnold D.D.P., Brown N.M., Gonzalez K.M. (2016). Hot topics, urgent priorities, and ensuring success for racial/ethnic minority young investigators in academic pediatrics. Int. J. Equity Health.

[61] Flores G., Mendoza F., Brimacombe M.B., Frazier W. (2020). Program evaluation of the research in academic pediatrics initiative on diversity (RAPID): Impact on career development and professional society diversity. Acad. Med..

[62] Flores G., Mendoza F.S., DeBaun M.R., Fuentes-Afflick E., Jones V.F., Mendoza J.A., Raphael J.L., Wang C.J. (2019). Keys to academic success for under-represented minority young investigators: Recommendations from the Research in Academic Pediatrics Initiative on Diversity (RAPID) National Advisory Committee. Int. J. Equity Health.

[63] Harawa N.T., Manson S.M., Mangione C.M., Penner L.A., Norris K.C., DeCarli C., Scarinci I.C., Zissimopoulos J., Buchwald D.S., Hinton L. (2017). Strategies for enhancing research in aging health disparities by mentoring diverse investigators. J. Clin. Transl. Sci..

[64] Hemming J., Eide K., Harwood E., Ali R., Zhu Z., Cutler J. (2019). Exploring professional development for new investigators underrepresented in the federally funded biomedical research workforce. Ethn. Dis..

[65] Jean-Louis G., Ayappa I., Rapoport D., Zizi F., Airhihenbuwa C., Okuyemi K., Ogedegbe G. (2016). Mentoring junior URM scientists to engage in sleep health disparities research: Experience of the NYU PRIDE Institute. Sleep Med..

[66] Mancuso C.A., Berman J.R., Robbins L., Paget S.A. (2019). What mentors tell us about acknowledging effort and sustaining academic research mentoring: A qualitative study. J. Clin. Ed. Health Prof..

[67] Manson S. (2016). Early-stage investigators and institutional interface: Importance of organization in the mentoring culture of today’s universities. AIDS Behav..

[68] Martina C.A., Mutrie A., Ward D., Lewis V. (2014). A sustainable course in research mentoring. Clin. Transl. Sci..

[69] Masterson-Creber R.M., Baldwin M.W., Brown P.J., Rao M.K., Goyal P., Hummel S., Dodson J.A., Helmke S., Maurer M.S. (2019). Facilitated peer mentorship to support aging research: A RE-AIM evaluation of the CoMPAdRE program. J. Am. Geriatr. Soc..

[70] Milburn N.G., Hamilton A.B., Lopez S., Wyatt G.E. (2019). Mentoring the next generation of behavioral health sciences to promote health equity. Am. J. Orthopsychiatry.

[71] Ofili E.O., Fair A., Norris K., Verbalis J.G., Poland R., Bernard G., Stephens D.S., Dubinett S.M., Imperato-McGinley J., Dottin R.P. (2013). Models of interinstitutional partnerships between research intensive universities and minority serving institutions (MSI) across the clinical translational science award (CTSA) consortium. Clin. Transl. Sci..

[72] Pfund C., Byars-Winston A., Branchaw J., Hurtado S., Egan K. (2016). Defining attributes and metrics of effective mentoring. AIDS Behav..

[73] Redmond N. (2020). Developing and Optimizing Your Mentoring Relationships.

[74] Shea J.A., Stern D.T., Klotman P.E., Clayton C.P., O’Hara J.L., Feldman M.D., Griendling K.K., Moss M., Straus S.E., Jagsi R. (2011). Career development of physician scientists: A survey of leaders in academic medicine. Am. J. Med..

[75] Shiramizu B., Shambaugh V., Petrovich H., Seto T.B., Ho T., Mokuau N., Hedges J.R. (2016). Leading by success: Impact of a clinical & translational research infrastructure program to address health inequities. J. Racial Ethn. Health Dispar..

[76] Snyder-Mackler L. (2015). 46th Mary McMillian Lecture: Not Eureka. Phys. Ther..

[77] Sorkness C.A., Pfund C., Ofili E.O., Kolawole S.O., Vishwanatha J.K. (2017). A new approach to mentoring for research careers: The National Research Mentoring Network (NRMN). BMC Proc..

[78] Stamatakis K.A., Norton W.E., Stirman S.W., Melvin C., Brownson R. (2013). Developing the next generation of dissemination and implementation researchers: Insights from initial trainees. Implement. Sci..

[79] Stoff D.M. (2019). Enhancing diversity and productivity of the HIV behavioral research workforce through research education mentoring programs. AIDS Behav..

[80] Sutton M.Y., Lanier Y.A., Willis L.A., Castellanos T., Dominguez K., Fitzpatrick L., Miller K.S. (2013). Strengthening the network of mentored, underrepresented minority and leaders to reduce HIV-related health disparities. Am. J. Public Health.

[81] Sweeney C., Schwartz L.S., Toto R., Merchant C., Fair A.S., Gabrilove J.L. (2017). Transition to independence: Characteristics and outcomes of mentored career development (KL2) scholars at clinical and translational science award institutions. Acad. Med..

[82] Teruya S.A., Bazargan-Hejaxi S., Mojtahedzadeh M., Doshi M., Russell K., Parker-Kelly D., Friedman T.C. (2013). A review of programs, components and outcomes in biomedical research faculty development. Int. J. Univ. Teach. Fac. Dev..

[83] Thorpe R.J., Vishwanatha J.K., Harwood E.M., Krug E.L., Unold T., Boman K.E., Jones H.P. (2020). The impact of grantsmanship self-efficacy on early state investigators of the National Research Mentoring Network Steps toward Academic Research (NRMN STAR). Ethn. Dis..

[84] Varkey P., Jatoi A., Williams A., Mayer A., Ko M., Files J., Blair J., Hayes S. (2012). The positive impact of a facilitated peer mentoring program on academic skills of women faculty. BMC Med. Educ..

[85] Velasquez G.E., Huaman M.A., Powell K.R., Cohn S.E., Swaminathan S., Outlaw M., Schulte G., McNeil Q., Currier J.S., Del Rio C. (2019). Outcomes of a career development program for underrepresented minority investigators in the AIDS clinical trials group. Open Forum Infect. Dis..

[86] Vermund S.H., Hamilton E.L., Griffith S.B., Jennings L., Dyer T.V., Mayer K., Wheeler D. (2018). Recruitment of underrepresented minority researchers into HIV prevention research: The HIV prevention trials network scholars program. Aids Res. Hum. Retrovir..

[87] Vishwanatha J.K., Jones H.P. (2018). Implementation of the steps toward academic research (STAR) fellowship program to promote underrepresented minority faculty into health disparity research. Ethn. Dis..

[88] Zambrana R.E., Ray R., Espino M.M., Castro C., Cohen B.D., Eliason J. (2015). Don’t Leave Us Behind: The importance of mentoring for underrepresented minority faculty. Am. Educ. Res. J..

[89] Nowell L., White D.E., Benzies K., Rosenau P. (2017). Exploring mentorship programs and components in nursing academia: A qualitative study. J. Nurs. Educ. Pract..

[90] Yun J.H., Baldi B., Sorcinelli M.D. (2016). Mutual mentoring for early career and underrepresented faculty: Model, research, and practice. Innov. High. Educ..

[91] Potter D.R., Tolson D. (2014). A mentoring guide for nursing faculty in higher education. Int. J. Caring Sci..

[92] Tourigny L., Pulich M. (2005). A critical examination of formal and informal mentoring among nurses. Healt. Care Manag..

[93] Huggett K.N., Borges J.J., Blanco M.A., Wulf K., Hurtubise L. (2020). A perfect match? A scoping review of the influence of personality matching on adult mentoring relationships—Implications for academic medicine. J. Contin. Educ. Health Prof..

[94] Allen T.D., Eby L.T., Lentz E. (2006). Mentoring behaviors and mentorship quality associated with formal mentoring programs: Closing the gap between research and practice. J. Appl. Psychol..

[95] Brown R.T., Daly B.P., Leong F.T. (2009). Mentoring in research: A developmental approach. Prof. Psychol. Res. Pract..

[96] Bean N.M., Lucas L., Hyers L.L. (2014). Mentoring in higher education should be the norm to assure success: Lessons learned from the faculty mentoring program, West Chester University, 2008–2011. Mentor. Tutor. Partner. Learn..

[97] Monaghan P. The Stress of Being a Minority Faculty Member. Chronicle of Higher Education, 65. https://www.chronicle.com/article/the-stress-of-being-a-minority-faculty-member/.

[98] Gumpertz M., Durodoye R., Griffin E., Wilson A. (2017). Retention and promotion of women and underrepresented minority faculty in science and engineering at four large land grant institutions. PLoS ONE.

[99] Tervalon M., Murray-Garcia J. (1998). Cultural humility versus cultural competence: A critical distinction in defining physician training outcomes in multicultural education. J. Health Care Poor Underserved.

[100] Waters A., Asbill L. Reflections on Cultural Humility. American Psychological Association. https://www.apa.org/pi/families/resources/newsletter/2013/08/cultural-humility.

[101] Chen Y., Watson R., Hilton A. (2016). A review of mentorship measurement tools. Nurse Educ. Today..

[102] Ng Y.X., Koh Z.Y.K., Yap H.W., Tay K.T., Tan I.H., Ong Y.T., Tan L.H.E., Chin A.M.C., Toh Y.P., Shivananda S. (2020). Assessing mentoring: A scoping review of mentoring assessment tools in internal medicine between 1990 and 2019. PLoS ONE.

[103] Huskins W.C., Silet L., Weber-Main A.M., Begg M.D., Fowler V.G., Hamilton J., Fleming M. (2011). Identifying and aligning expectations in a mentoring relationship. Clin. Transl. Sci..

